# A scoping review on the measurement of transnationalism in migrant health research in high-income countries

**DOI:** 10.1186/s12992-021-00777-2

**Published:** 2021-10-29

**Authors:** Ye Na Kim, Marcelo Urquia, Sarah Fredsted Villadsen, Lisa Merry

**Affiliations:** 1 Faculty of Nursing, University of Montreal, Montreal, Quebec Canada; 2 Manitoba Centre for Health Policy, Department of Community Health Sciences, Rady Faculty of Health Sciences, University of Manitoba, Winnipeg, Manitoba Canada; 3 Department of Public Health, Univeristy of Copenhagen, Copenhagen, Denmark; 4 SHERPA University Institute, West-Central Montreal CIUSSS, Montreal, Quebec Canada; 5 InterActions Centre de recherche et de partage des savoirs, CIUSSS du Nord-de-l’île-de-Montréal, Montreal, Quebec Canada

**Keywords:** Transnationalism, Cross-border ties, Migration, Health, Well-being, Operationalization and measurement

## Abstract

**Background:**

Migrants commonly maintain transnational ties as they relocate and settle in a new country. There is a growing body of research examining transnationalism and health. We sought to identify how transnationalism has been defined and operationalized in migrant health research in high income countries and to document which populations and health and well-being outcomes have been studied in relation to this concept.

**Methods:**

We conducted a scoping review using the methodology recommended by the Joanna Briggs Institute (JBI). We searched nine electronic databases; no time restrictions were applied. Studies published in English or French in peer-reviewed journals were considered. Studies were eligible if they included a measure of transnationalism (or one of its dimensions; social, cultural, economic, political and identity ties and/or healthcare use) and examined health or well-being.

**Results:**

Forty-seven studies, mainly cross-sectional designs (81%), were included; almost half were conducted in the United States. The majority studied immigrants, broadly defined; 23% included refugees and/or asylum-seekers while 36% included undocumented migrants. Definitions of transnationalism varied according to the focus of the study and just over half provided explicit definitions. Most often, transnationalism was defined in terms of social connections to the home country. Studies and measures mainly focused on contacts and visits with family and remittance sending, and only about one third of studies examined and measured more than two dimensions of transnationalism. The operationalization of transnationalism was not consistent and reliability and validity data, and details on language translation, were limited. Almost half of the studies examined mental health outcomes, such as emotional well-being, or symptoms of depression. Other commonly studied outcomes included self-rated health, life satisfaction and perceived discrimination.

**Conclusion:**

To enhance comparability in this field, researchers should provide a clear, explicit definition of transnationalism based on the scope of their study, and for its measurement, they should draw from validated items/questions and be consistent in its operationalization across studies. To enhance the quality of findings, more complex approaches for operationalizing transnationalism (e.g., latent variable modelling) and longitudinal designs should be used. Further research examining a range of transnationalism dimensions and health and well-being outcomes, and with a diversity of migrant populations, is also warranted.

**Supplementary Information:**

The online version contains supplementary material available at 10.1186/s12992-021-00777-2.

## Introduction

Transnationalism is recognized as a dominant feature in migrants’ lives, and researchers have indicated that there are complexities and ambiguities associated with its conceptualization [1–4]. Multiple definitions of transnationalism exist, and in some instances it is considered more of a framework rather than a concept [4]. ‘Transnationalism from below’ (rather than ‘from above’, at the government and corporate levels), refers to transnationalism at the micro level, mainly individuals, and represents the assorted ways that international migrants (e.g., immigrants, refugees) continue to maintain connections with their country of origin, and/or other countries of significance after resettling in a new country [3]. These include social, cultural, economic, and political activities and interactions that take place in the host country, home country, and/or through various methods of communication across borders [3, 5]. Transnational ‘ways of being’ may involve the exchange of information, material goods and money, civic and political engagement, the maintenance of emotional connections with family members and friends, and the use of services, including healthcare [6–8]. Transnationalism also involves a more subjective component, ‘ways of belonging’, which encompasses identities, consciousness, and emotions that may precede and/or may be an outcome of transnational transactions [6, 7]. This may include, for example, a migrant’s desire to return to their country of origin or a continued sense of attachment to their homeland all the while remaining in their new destination country [9]. Identities, interactions and exchanges are not static, and shift over time.

Though the concept of transnationalism and its place and impact on migrants’ lives have been discussed and studied within sociology and anthropology for decades [3–5, 7], it is only more recently that this concept has been considered in migrant health research [8]. Transnationalism is increasingly recognized as affecting the health and well-being of migrants both positively and negatively [7, 8, 10–15]. For example, maintaining regular contact with family and friends in the home country has been associated with emotional well-being and life satisfaction [15]. It has been proposed that this may be due to the sense of connection and cohesion and emotional support that can result from these long-distance relationships [15]. In contrast however, sending remittances, although shown to elicit positive emotions as it can improve the lives of family back home, has been found to be related to poorer mental health due to the financial strain that it can cause [16, 17]. Similarly, family separation, and transnational parenting and caregiving have been associated with stress [17–20], which can affect not only mental health but also can contribute to chronic diseases. Transnationalism has therefore been considered a risk factor as well as a source of resilience for migrant health and well-being [15, 21, 22]. Research on transnationalism and health has mostly been studied using qualitative methods, however, there is also a growing body of quantitative research that aims to illustrate the strength and direction of the relationships between transnationalism and health and well-being. A key issue for the latter, is how best to measure the concept of transnationalism and its various dimensions (i.e., social, cultural, economic, political and affective/identity ties and/or healthcare use).

To our knowledge, there is no review that provides a comprehensive overview of how transnationalism has been measured and studied in relation to health and well-being. The current review sought to systematically identify and describe questions/tools and approaches being used to measure transnationalism within migrant health research in high income countries, including how transnationalism has been defined and operationalized, and to document which populations and health outcomes have been studied in relation to this concept. The overall goal was to provide researchers an inventory of existing questions and approaches available for measuring transnationalism. The intention was also to identify shortcomings of research in this field in order to inform future work.

### Research questions

1. How has transnationalism been defined and measured in migrant health research in high-income countries?
2. Which populations (migrant groups and host countries) and health and well-being outcomes have been examined in this body of research?

## Methods

We followed the methodology for scoping reviews recommended by the Joanna Briggs Institute (JBI) [23]. A scoping review is used to address broad questions and to provide an overview on a particular topic. The questions are usually based on the ‘population’, ‘concept’, and ‘context’ elements, rather than the typical ‘population, intervention, comparator, and outcome’, which are used to guide traditional systematic reviews. The purpose of a scoping review is to offer a foundation upon which future reviews or studies can build either by identifying research gaps and/or by describing and clarifying key concepts, tools, and characteristics in a specific area of inquiry. A scoping review was therefore deemed suitable since our objective was to summarize how ‘transnationalism’ (concept) has been measured and studied in ‘health research’ (context) conducted with ‘migrants living in high-income countries’ (population), and to provide information that would assist researchers in planning their approach for measuring transnationalism in future studies.

### Search strategy

The search strategy was developed in consultation with a university librarian and with the support of a research assistant. The search was conducted on February 19th 2020 by YK in MEDLINE, Global Health, PsycINFO, Embase, CINAHL, Anthropology Plus, Sociological Abstracts, ProQuest Central, and Web of Science; the “alerts function” was used to identify relevant publications after this date and an update search was conducted on July 12th 2020. The search strategy included a list of index/subject terms (e.g. MeSH terms in MEDLINE) as well as keywords related to, or describing transnationalism, physical and mental health, well-being, health behaviours, and social support. Social support was included in the search strategy as it has been shown to be a common outcome in relation to transnationalism. The search strategy was tailored to each database. Test searches were conducted in MEDLINE and CINAHL to refine the terms and keywords. For ‘transnationalism’ we did not include terms specific to its dimensions, such as ‘remittance sending’ and ‘distance parenting’, or related terms, such as ‘family separation’, because the number of hits was too unwieldly. Keywords were searched in the titles, abstracts, subject fields and keywords. Based on our language capabilities, we limited our search to studies published in English and French. There were no time restrictions. We also hand-searched the included studies’ reference lists using the same criteria. Details for each database search are provided in Additional file 1.

### Eligibility criteria

We included research studies that had at least one quantitative measure of transnationalism (or one of its dimensions) and that examined health or well-being in migrants living in a high-income country; quantitative and mixed-methods’ studies were therefore both eligible for inclusion. Literature reviews, abstracts and commentaries were not considered as they were deemed less relevant sources for addressing the objectives of the review. We only included research published in peer-reviewed journals since we expected there to be considerable overlap between the studies reported in journal articles and the gray literature (books and dissertations). We also anticipated that exclusion of the latter would not change the conclusions of the review. “Migrant” was defined as anyone born outside the host country, including individuals without legal status; migration could have been for any reason (forced, economic, family, and/or for educational purposes), and could have been temporary or permanent [24]. High-income countries included Canada, the United States, Australia, New Zealand and European countries.

Examples of health and well-being outcomes that we searched for included those related to physical or mental health, as evaluated by self-report or standardized questionnaires (e.g., Kessler Psychological Distress Scale); lifestyle behaviors; subjective or emotional well-being, assessed using various questionnaires/research tools; and measures of quality of life, social support and relationships. The health or well-being outcome could have also been examined qualitatively. Since the temporal sequence of association cannot always be confirmed, we included studies that examined “predictors” of transnationalism as long as the predictors were health or well-being related.

We included studies that examined transnational families, as long as they included at least one outcome related to health or well-being of migrants living in a high-income country. Transnational families are generally defined in the literature as nuclear families that live across borders, such as parents who migrate internationally while leaving their children in the care of relatives in the country of origin, or migrants who experience cross-border separation from spouses or partners [25]. Although we did not explicitly search for studies that focused on specific dimensions of transnationalism (e.g., remittance sending, distance parenting), we did include them if they were identified through our search. While transnationalism encompasses maintenance of language and cultural practices and traditions, for the purpose of this review, we did not include studies that solely examined language and culture in relation to health or well-being (i.e., there had to be some mention of transnational or cross-border interactions or activities). Studies that focused on medical tourism in general (i.e., travelling abroad to obtain healthcare) were also excluded, but those that examined transnational healthcare use among migrants living in one of the countries listed above, were considered eligible.

### Data extraction, analysis and synthesis

All citations were downloaded and managed using Endnote X8 software (version 18.0.1.12636). YK selected potentially eligible literature by first screening titles and abstracts. Duplicates and papers that clearly did not meet the eligibility criteria were removed at this step. All remaining articles were then retrieved and reviewed to determine eligibility. YK was responsible for the initial review and selection and when eligibility was uncertain, a decision was made via a discussion with LM. LM read and confirmed all of the included literature.

YK extracted and entered data for all included literature into an Excel database. LM independently reviewed each article and verified the data extraction. Extracted data included: the year and language of the publication; the objective and general findings of the study in relation to transnationalism; the host country or countries where the study took place; the research design; the data collection method; the description of the migrant population; the sample size; and the health and/or well-being outcome(s) examined. We also recorded descriptions of the tools used to measure transnationalism, including definitions of transnationalism, the type of transnational ties measured, the response format, the number of items, the language(s) of the tools, and information about validity and reliability; relationships between transnationalism and the outcomes were noted as well in order to present additional data about the measurement of transnationalism. Although it is not required for scoping reviews, we also conducted a general appraisal of the methodological quality of the studies using the Mixed Methods Appraisal Tool (MMAT) Version 2018 [26]. We conducted these appraisals with the purpose of providing more contextual information regarding how transnationalism and health and well-being had been studied; no studies were excluded based on these assessments. The MMAT comprises two screening questions and a set of five questions on methodological rigour that vary depending on the type of research being assessed (e.g., quantitative, mixed-methods). Each item requires a ‘yes, no, or can’t tell’ response, and has a space for comments. The items are not meant to be calculated into a score, but rather they provide a general appreciation of the methodological strengths and weaknesses of a study. All information gathered was compiled and synthesized into summary tables and narrative text.

## Results

**Fig. 1:**
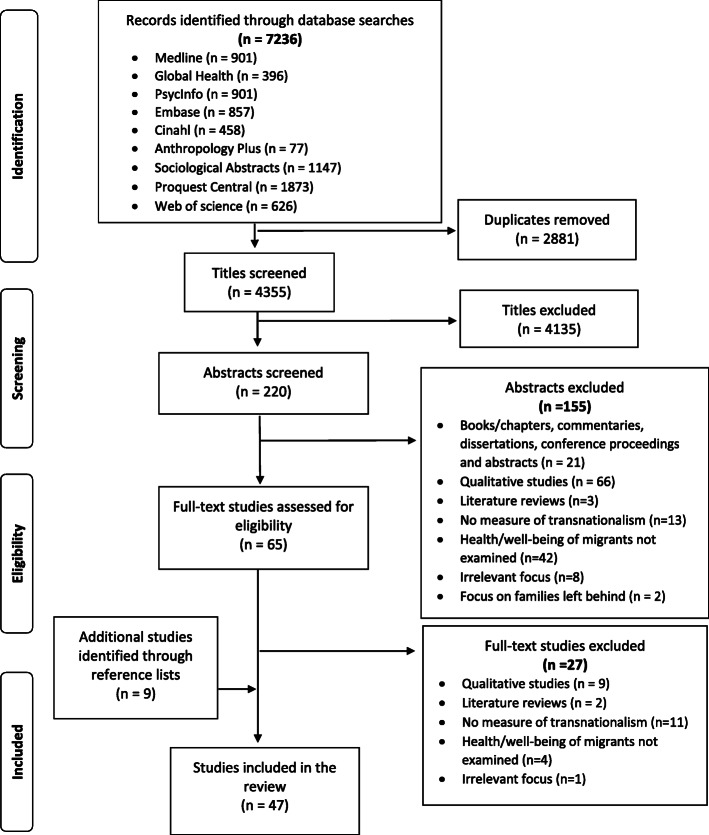
PRISMA flow diagram

The database searches yielded 7236 records, of which 2881 were duplicates. A total of 65 papers were retrieved for full-text review (Fig. 1). At this step, 27 papers were excluded; nine were qualitative studies, two were literature reviews, 11 had no measures of transnationalism, four did not examine health or well-being of migrants, and one paper had an irrelevant focus. Forty-seven studies fully met the eligibility criteria and were included in the review.

### Study characteristics

Detailed descriptions of the included studies are reported in Appendix I (Additional file 2); a summary is presented in Table 1. The studies were published between 2004 and 2020 and all were published in English (no studies published in French were identified). The majority of the studies were quantitative, cross-sectional designs (*n* = 38, 80.9%). Twenty-six studies (55.3%) were quantitative studies that used population-based surveys [21, 22, 27–50]. Four studies used a mixed-methods design (8.5%), all of which had an emphasis on the qualitative component; one involved using interview data to develop a quantitative questionnaire, which was then used to generate data to complement the qualitative data [51]; two used quantitative questionnaires to supplement qualitative findings [52, 53] and one study used interviews to gain a more in-depth understanding of survey results [54]. In almost half of the research (*n* = 23, 48.9%), at least one of the primary objectives was to examine transnationalism and outcomes such as health status, subjective well-being, and/or mental health, including feelings of loneliness [13, 21, 22, 29, 30, 36–41, 44–46, 50, 55–62]. The relationships between transnationalism and socioeconomic factors that are important to well-being, like financial strain, job outcomes and housing issues (e.g. houses being too small, or difficulties finding lodging), were a main interest in five studies [40, 52, 60, 63, 64]. For ten studies (21.3%), the objective was to examine transnationalism and other types of well-being outcomes, for example discrimination, integration, lifestyle behaviours, and social relationships [27, 28, 32, 33, 42, 43, 65–68]. A handful of studies (*n* = 6, 12.8%) focused on transnational healthcare use and associated factors, such as health status, and/or healthcare experiences in the host country [31, 35, 47–49, 54]. In five studies, measuring the associations between transnationalism and health or well-being factors was not a principal aim of the research [34, 51, 53, 69, 70].

**Table 1:** Descriptive summary of studies

Descriptor	Studies *N* = 47, n(%)
Year of publication	
Jan 2004 – Dec 2010	7(14.9%)
Jan 2011 – Dec 2019	37(78.7%)
Jan 2020 – Aug 2020	3(6.4%)
Study design	
Cross-sectional survey	38(80.9%)
Longitudinal survey^a^	5(10.6%)
Mixed methods	4(8.5%)
Location of study (migrant resettlement country)^b^	
North America	
United States	23(48.9%)
Canada	5(10.6%)
Unspecified	1(2.1%)
Europe	
Netherlands	10(21.3%)
France	4(8.5%)
Ireland	2(4.3%)
Denmark	1(2.1%)
Finland	1(2.1%)
Germany	2(4.3%)
Spain	1(2.1%)
Portugal	1(2.1%)
Italy	1(2.1%)
United Kingdom	2(4.3%)
Unspecified	1(2.1%)
New Zealand	2(4.3%)
Australia	1(2.1%)
Other countries	2(4.3%)
Migrants’ region of origin^b^	
Sub-Saharan Africa	12(25.5%)
Africa, unspecified	4(8.5%)
Northern Africa / Middle East / Turkey	7(14.9%)
Latin America and ‘Black’ Caribbean	
Mexico	12(25.5%)
Central America	6(12.8%)
South America	4(8.5%)
Caribbean (Cuba, Puerto Rico, Dominican Republic)	10(21.3%)
Latin America, unspecified	12(25.5%)
‘Black’ Caribbean	4(8.5%)
Caribbean, unspecified	3(6.4%)
Asia	
Southeast Asia	4(8.5%)
South Asia	3(6.4%)
East Asia (China, Korea)	9(19.1%)
Unspecified	4(8.5%)
Europe (mostly Eastern Europe)	9(19.1%)
Australia/New Zealand	1(2.1%)
North America	3(6.4%)
Unspecified	3(6.4%)
Sample size (quantitative)	
50–500	13(27.7%)
501–1000	9(19.1%)
1001–2000	15(31.9%)
2001–5000	5(10.6%)
5001–10,000	3(6.4%)
10,001-20,000	1(2.1%)
> 20,000	1(2.1%)
Migrants’ generation^b^	
1st generation migrants	47(100%)
2nd generation migrants	12(25.5%)
≥ 3rd generation migrants	3(6.4%)
Migrants’ status^b^	
Immigrants^c^	46(97.9%)
Refugees and/or Asylum-seekers^d^	11(23.4%)
Undocumented migrants	17(36.2%)

^a^One study conducted a cross-sectional analysis

^b^A study may be counted in more than one category so percentages do not add to 100%

^c^Broadly defined, including those who immigrated through economic, family and business categories; most studies did not specify the immigrant categories and only described the population as “immigrants”

^d^Includes those who had an asylum history but obtained residency status

Almost half of the studies (*n* = 23, 48.9%) were conducted in the United States and many of these (*n* = 18) focused on migrants originating from Latin America (mainly Cuba, Puerto Rico, Mexico and countries in Central America) [22, 28, 29, 32, 34, 41–47, 49, 52, 60, 61, 69, 70] and/or East/South-East Asia (China, Korea, Philippines, and Vietnam) (*n* = 4) [34, 43, 54, 55]. Ten studies (21.3%) were conducted in the Netherlands, six focused on migrants from Africa; specifically, Angola (*n* = 4) [57, 59, 63, 67], Nigeria (*n* = 3) [57, 62, 67], and Ghana (*n* = 1) [56]. Three of the studies conducted in France (*n* = 4, 8.5%) also focused on migrants from sub-Saharan Africa [21, 27, 37]. Among the Canadian studies (*n* = 5, 10.6%), two had a mix of migrants from different regions of the world [30, 31], one focused on migrants from Korea [53], another focused on Sudanese refugees [64], and the other, examined migrants from the Philippines [40]. Eastern Europeans, including migrants from the former Yugoslavia, Poland, Romania and Russia were mainly studied in Europe (*n* = 6) [33, 35, 38, 50, 66, 68].

All of the studies included first generation migrants (this was an inclusion criteria); 12 (25.5%) also included second generation migrants [21, 27, 32, 39, 41, 42, 44, 48, 49, 52, 60, 68]. Three of the latter also had third and/or later generation migrants [39, 42, 49]. The study populations were often described as “immigrants” without specifying the immigration status of the participants. Refugees and asylum-seekers (or those with an asylum-seeking history) were included in the samples of 11 studies (23.4%) [30, 32, 34, 46, 57, 59, 62–64, 67, 68], while undocumented migrants were included in 17 studies (36.2%) [22, 37, 41, 47, 49, 52, 54, 56–59, 61–63, 66, 67, 70]. One study included Ingrian Finnish returnees [35]. Studies that included refugee and/or undocumented migrants mostly took place in the Netherlands and the United States.

All of the research was conducted with adult migrants; a subset of these focused on migrants who were parents (*n* = 9, 19.1%) [52, 55–57, 59, 60, 62, 63, 67], and three of these specifically focused on mothers [52, 55, 60]. Four studies (8.5%) focused on older migrants [36, 42, 44, 65]. Other specific populations studied included nurses [51], Sudanese refugee men [64], low-income women who had accessed social services/programs [61] and migrants who had visited healthcare facilities, many of whom were living with HIV or chronic hepatitis B [37]. None of the publications on transnational families examined outcomes among family members back in the country of origin [37, 50, 55–57, 59–63, 67].

### Definitions and measures of transnationalism

Definitions of transnationalism used in each study can be found in Table 2. Of the 47 studies, 25 (53.2%) explicitly and clearly defined transnationalism [13, 21, 22, 27, 29, 30, 33, 36, 38, 39, 41–45, 52–54, 57, 59, 62, 63, 65, 67, 68]. The majority of studies did however, include a literature review relevant to the purpose of the study which provided some conceptual foundation. In general, definitions used varied accordingly with the focus of the study. Most often, authors described transnationalism as migrants’ ongoing social, material, economic, cultural and emotional connections and activities across international borders; the “patterns of living that span across countries” [22, 28, 29, 32, 38, 39, 41, 43–45, 58]. Equally common was a definition that primarily focused on, or that emphasized, the social relations and multiple social fields that migrants build between their countries of origin and the host country [13, 21, 27, 30, 33, 34, 37, 42, 65, 68]. Some studies examined a specific transnational dimension including remittance sending (*n* = 4, 8.5%) [40, 46, 51, 64], cross-border parenting or relationships between spouses or partners (*n* = 10, 21.3%) [50, 55–57, 59–63, 67], and transnational belonging (n = 1, 2.1%) [36], and so did not directly refer to the concept of transnationalism. Instead, definitions of the specific transnational dimension studied were provided. Remittances were described as money sent by migrants to relatives in their country of origin; one study also included money sent to family or friends living in the same destination country but a separate household, as part of the definition of remittance sending [46]. Definitions of cross-border use of healthcare included migrants’ use of healthcare *from* (in the form of medication, remedies, or information), or *in* the country of origin (in the form of services), without explicitly being situated within a framework of transnationalism [31, 35, 47–49, 53, 54]. However, two studies [53, 54] described how transnational healthcare use is closely related to other types of transnational ties and that it adds another dimension to the concept of transnationalism.

**Table 2:** Definitions and measures of transnationalism^a^

	1st author, year (reference #)	Study’s definition of Transnationalism	Administration Method	Language^b^, Translation^c^	No. of items	Response format Y/N = Yes / No format	Transnationalism dimension assessed						Quality appraisal Internal consistency (C)^d^
							Social	Cultural	Economic	Political	Attitude/Identity	Healthcare	
1	Afulani, 2015 [27]	Process by which migrants forge and sustain social relations that link their country of origin and destination country, or the maintenance of activities that require regular contact across borders	In-person interview	French (SL) Unspecified mother tongue (O)^e^	9	Y/N (then scored out of 9)	X	X	X	X			C = 0.60
	Afulani, 2016 [21]												
2	Alcantara, 2015 [29]	Frequent and enduring social, economic, political, or cultural ties between two or more countries	In-person interview	Eng (SL) Spa (?)^f^	2	Continuous	X		X				
	Alcantara, 2015 [28]				2	Y/N & continuous	X		X				
	Gelatt, 2013 [34]	The process by which immigrants build social fields that link together their country of origin and destination country		Eng (SL) Unspecified mother tongue (?)^f^	4	Y/N & categorical	X		X		X		
	Torres, 2016 [22]	The dual involvement of migrants in social life, religious and cultural practices, healthcare and social service systems, and political activities and entrepreneurial ventures across nation-state boundaries		Eng (SL) Spa (?)^f^	2	Y/N & categorical	X		X				
	Torres, 2019 [43]	Immigrants’ contact with family and friends in their country of origin through long-distance communication, remittance-sending, political participation and return visits			2	Y/N & categorical	X						
3	Ambugo, 2016 [46]	Remittances: money sent by migrants to family / friends living abroad or in the same destination country but in separate household	Interview	Eng	2	Y/N & continuous			X				
4	Amoyaw, 2016 [30]	Redefining, reproducing, and maintaining ties with relatives and friends in the country of origin; remittances are the oldest and most popular form of transnational behavior	Interview	Eng (SL) Unspecified mother tongue (?)^f^	3	Y/N & continuous (converted to a categorical variable)		X	X				
	Calvasina, 2015 [31]	Medical transnationalism: returning to the country of origin for the sole purpose of obtaining healthcare; transnational dental care = seeking dental care across national borders, and can involve opportunistic dental visits while traveling to country of origin			1	Y/N						X	
	Shooshtari, 2014 [40]	Remittances: money sent by immigrants to family members in their country of origin			2	Y/N & continuous			X				
5	Araujo Dawson, 2010 [69]	Not provided	In-person self-completed survey, assistance provided as needed	Eng (SL) Spa (B)^f^	21	Average score computed	X	X	X	X			C = 0.84
	Murphy, 2004 [13]	Maintenance of occupations / activities that necessarily require regular social contact over time across borders and / or cultures	In-person or online self-completed survey	Eng		6-point Likert scale (0–5)							C = 0.87
6	Araujo Dawson, 2018 [32]	Back and forth migration (i.e. familial, political, economic, and social activities) between destination and country of origin	Telephone survey	Eng (SL) Spa (?)	10	Y/N (then scored out of 10)	X		X	X	X		
7	Burholt, 2016 [65]	Process by which migrants forge and sustain multi-stranded social relations through the creation of cross-border networks	In-person interview	Eng (SL) Punjabi, Gujarati, Hindi, Mandarin, Bengali, Somali, Yoruba, Urdu (B)	13	Categorical & continuous (converted into a categorical variable using latent variable modelling)	X		X				
8	Chang, 2018 [55]	“Kirogi family”: a transnational living arrangement whereby one parent accompanies their child (ren) to an English-speaking country for educational purposes while the other remains in Korea to support them financially	Online or mailed survey	Eng (SL) Korean (B)	1	Y/N	X						
9	De Jesus, 2013 [47]	Transnational healthcare use: seeking health care outside the US to overcome access barriers to healthcare	Telephone survey	Eng (SL) Spa (?)	2	Y/N					X	X	
10	Dito, 2017 [56]	Transnational parents: those that migrate and leave their child (ren) in the country of origin in the care of another family member, while also participating in raising their children across borders	In-person interview	Eng (?)^g^ or Dut (?)^g^	1	Y/N	X						
	Haagsman, 2018 [63]	Transnational families: when men / women migrate without their nuclear families (i.e. parents migrate while children remain in the country of origin in the care of others)		Eng (?)^f,g^ or Dut (?)^g^ Por (B)^f^	2	Y/N & categorical	X						
	Haagsman, 2014 [67]				2	Y/N & categorical	X		X				
	Haagsman, 2015 [57]				1	Y/N	X						
	Mazzucato, 2017 [59]				2	Y/N	X		X				
	White, 2019 [62]	Transnational families: families who live apart but retain a sense of collective welfare and identity across national borders		Eng (?)^f,g^ or Dut (?)^g^	2	Y/N & categorical	X		X				
11	Djundeva, 2020 [33]	Migrants actively construct multiple social relations that bring the country of origin and destination country together	Online survey & computer-assisted personal interview	Dut (SL) Polish (?)	5	Y/N & continuous	X	X	X		X		
	Van den Broek, 2017 [50]	Transnational relationships: migrants with a partner living abroad			2	Categorical	X						
12	Flippen, 2015 [70]	Process of exchange, connection, and mobility across national borders	In-person interview	Eng (SL) Spa (?)	2	Y/N & categorical	X				X		
13	Gherghina, 2020 [66]	Return intentions: the result of integration in the destination country, transnationalism, and the interaction between the two Transnationalism: attachment to the country of origin	Online survey	Not specified	1	Y/N					X		
14	Greder, 2009 [52]	Means by which migrants maintain connections with their country of origin while continuing to develop relationships in destination communities	In-person interview	Eng (SL) Spa (T)^f^	5	Y/N (then scored out of 5 and then converted to a categorical variable)	X	X	X				
15	Horn, 2020 [58]	A phenomenon where migrants, through daily activities, forge and sustain multi-stranded social, economic, and political relations that link together their societies of origin and settlement	In-person interview or self-report survey	Not specified: Maybe Spa (SL)^g^	6	Y/N & categorical	X	X	X		X		
16	Humphries, 2009 [51]	Remittances: money sent by migrants to their families in the country of origin	Mailed survey	Eng	3	Y/N, continuous & categorical			X				
17	Jang, 2017 [54]	The extent to which migrants maintain links to their homeland; Medical transnationalism: receiving medical care from the country of origin (may not require migrants to actually visit)	Self-report survey	Eng	2	Y/N & categorical	X					X	
18	Johnson, 2008 [64]	Remittances: money / goods sent to relatives in the country of origin	Self-report survey	Eng (SL) Ara (T)	3	Continuous & categorical			X				
19	Kempainnen, 2018 [35]	Cross-border healthcare: returning to the country of origin for healthcare	In-person interview	Finnish (SL) Russian (?)^f^	1	Y/N						X	
20	Klok, 2017 [36]	Transnational belonging: sense of belonging directed to own group in the country of origin	In-person interview	Dut (SL) Turkish (O)^f^ Moroccan (O)^f^ Ara/Darijia (O)^f^ Tarafit (O)^f^	6	Categorical (but each item was treated as a score)	X				X	X	cultural identity: C = 0.67; feelings of loss: C = 0.70
21	McCabe, 2017 [60]	Transnational parenting: separation of families, particularly of mothers from children	In-person interview	Eng (SL) Spa (?)^f^	1	Y/N & categorical	X						
22	Miranda, 2005 [61]	Transnational separation: parents (mothers) migrating and leaving their child (ren) behind	In-person interview	Eng (SL) Spa (?)^f^	1	Y/N	X						
23	Nielsen, 2012 [48]	Patient mobility: patients’ deliberate movements across international borders to seek planned healthcare	Computer-assisted telephone interview or online survey	Eng (?)^g^ or Danish (?)^g^ Turkish (B)^f^	2	Y/N						X	
24	Pannetier, 2017 [37]	Cross-border family separation and transnational ties particularly in the form of financial transfers	In-person interview	French (SL) Unspecified mother tongue (O)^e^	2	Y/N & categorical	X						
25	Razum, 2019 [38]	Migrants have resources at their disposition that relate to transnational ties / practices that connect them to their country of origin; transnationalism may vary in degree and vary across social life, familial, economic, sociocultural, or political spheres	Interview	Not specified: Maybe German (SL)^g^	7	Y/N (then scored out of 7 and then converted to a categorical variable)	X	X	X		X		
26	Samari, 2016 [39]	Social, material, and emotional support migrants exchange with their countries of origin	In-person interview	Eng (SL) Ara (B)^f^	21	Y/N & categorical (Average score then computed for each dimension)	X	X		X	X		cross-border attitudes: C = 0.72; media consumption: C = 0.63; social ties: C = 0.65; community organizations: C = 0.84
27	Snel, 2006 [68]	Transnational migration: pattern of migration in which migrants settle in a new country while maintaining ongoing social connections with their country of origin; people living their lives across international borders	In-person interview	Preferred language of migrants from Morocco, Dutch Antilles, Iraq, former Yugoslavia, Japan, and the United States (?)^f,g^	17 + 50 statements on identity	Y/N & categorical (Scores were also calculated)	X	X	X	X	X		sense of belonging to home country: C = 0.81; norms and values of home country: C = 0.86; norms and values of international diaspora: C = 0.84
28	Su, 2012 [49]	Cross-border health utilization: physically seeking healthcare across borders	Telephone interview	Eng Spa (?)	2	Y/N						X	
29	Torres, 2013 [41]	Participation of activities in or related to migrants’ country of origin, may include a range of dimensions (i.e. economic, political, cultural)	Telephone survey	Eng (SL) Spa (?)^f^	5	Y/N	X		X				
30	Torres, 2018 [42]	Migrants maintaining social connections to family / friends both locally and in their countries of origin	In-person interview	Eng (SL) Spa (?)	1	Categorical	X						
	Torres, 2016 [44]	Social, political, economic, and cultural spaces formed and reworked by migrants in the destination and country of origin, and the flow of capital, goods, ideas, and individuals within these spaces			2	Categorical	X						
31	Vaquera, 2011 [45]	The development of networks, activities, and patterns of living that span origin and destination countries	Telephone survey	Eng (SL) Spa (T) Haitian Creole (T)	8	Categorical	X	X	X		X		
32	Wang, 2015 [53]	Medical transnationalism: migrants’ efforts to maintain and make use of transnational ties with the country of origin in managing their health and well-being	Interview	Eng (SL) Korean (T)^f^	8	Y/N	X	X	X				

^a^Studies that used the same measure (or parts of the same measure) are grouped together; 32 measures were used across all of the studies

^b^*Eng* English, *Ara* Arabic, *Spa* Spanish, *Dut* Dutch, *Por* Portuguese

^c^*SL* source language (i.e., the original language of the measure), *T* simple direct translation, *B* Translation & back-translation, *O* Oral translation,? = translation process unclear

^d^Chronbach’s alpha

^e^Professional interpreter

^f^Bilingual/bicultural interviewer

^g^Unclear source language

The descriptions of the questions, survey instruments, and rating scales on transnationalism as reported in the articles are also presented in Table 2. A number of studies used the same questionnaire/instrument (or parts of it) to measure transnationalism, for a total of 32 measures across the studies. The majority of these were developed in English (*n* = 21) [13, 22, 28–32, 34, 39–47, 49, 51–55, 60, 61, 64, 65, 69, 70], other source languages included French [21, 27, 37], Dutch [33, 36, 50], and Finnish [35]; for some, the source language was not clear [48, 56, 57, 59, 62, 63, 67, 68]. Three studies (6.4%) did not provide any information on language [38, 58, 66]. In many studies, the transnationalism measure was translated; nine studies (19.1%) reported doing translation and back-translation of their measure [39, 48, 55, 57, 59, 63, 65, 67, 69]; four (8.5%) did simple, direct translation [45, 52, 53, 64]; and four (8.5%) used verbal translation via a professional interpreter or a bilingual interviewer [21, 27, 36, 37]. Twenty-one studies (44.7%) did not provide clear information about the translation process [22, 28–35, 40–44, 47, 49, 50, 60, 61, 68, 70], and for two other studies (4.3%), it was unclear on whether or not there was translation since the source language was not indicated (i.e., the participants were English speaking living in the Netherlands and it was not clear if the questions were initially developed in English or Dutch) [56, 62]. The most common languages the tools were translated to were Spanish (*n* = 16, 34.0%) [22, 28, 29, 32, 41–45, 47, 49, 52, 60, 61, 69, 70], Arabic or Turkish (*n* = 5, 10.6%) [36, 39, 48, 64, 68] and Portuguese (*n* = 4, 8.5%) [57, 59, 63, 67]. Two studies (4.3%) respectively, measured transnationalism in Korean [53, 55], and Polish [33, 50], while five studies (10.6%) translated their questions to other languages [35, 36, 45, 65, 68]; seven studies indicated “preferred language or “mother tongue” without specifying the languages [21, 27, 30, 31, 34, 37, 40]. More than 75% of the studies (*n* = 36) gathered transnationalism data via interview-administration of their measures, five of these clearly indicated that the interviews were done by telephone [32, 41, 45, 47, 49]. In seven studies, data were collected through self-completed surveys, mainly online or by mail [13, 51, 54, 55, 64, 66, 69]. Four studies (8.5%) offered a mix of interview and self-completion methods [33, 48, 50, 58].

The number of items measuring transnationalism ranged from one to 21, with the majority of studies (*n* = 34, 72.3%) using five items or less. Items required either a yes/no, or a numerical (e.g. number of visits made to the country of origin) response, or required respondents to select from options (e.g. sending remittances often or not very often). In most studies, the items were maintained as single variables, whereas in some studies they were combined to calculate scores or to create categories to indicate higher and lower levels of overall or specific dimensions of transnationalism [13, 21, 27, 36, 38, 52, 55]. In one study, 13 variables were used to generate four different categories of transnational family relationships using latent variable modelling [65]. In another study, in addition to a questionnaire, the researchers used an extensive two phase process involving 50 statements to measure transnational identity [68]. In phase one, participants were asked to respond four times to the set of statements in order to assess how much they identified with the host population, their ethnic group living in the host country, their diaspora living in other countries, and their country of origin, respectively. In phase two, they were asked to respond to each statement by placing two circles, one representing the host country, and the other their country of origin, as either overlapping or as separate from one another with varying spacing, in order to visually depict their degree of transnationalism. The results from the two phases were then used to generate various identity scores.

In terms of which dimensions of transnationalism were measured across the research, social (*n* = 36, 76.6%) and economic ties (*n* = 26, 55.3%) were the most frequently measured. Transnational attitudes/identity (*n* = 12, 25.5%) and cultural ties (*n* = 13, 27.7%) were measured less often; and transnational healthcare use (*n* = 7, 14.9%), and political ties (*n* = 7, 14.9%) were measured the least. One study [68] measured all the types of ties (i.e. social, cultural, economic, political, and attitudes) whereas fourteen studies (29.8%) measured three to four dimensions, twelve studies (25.5%) measured two, and 20 (42.6%) measured one. A summary of how items were operationalized can be found in Appendix II (Additional file 2).

Social transnational ties were mainly measured by assessing whether respondents made return visits (*n* = 23, 48.9%) [13, 21, 22, 27–29, 32–34, 36, 38, 39, 41, 43–45, 52, 53, 58, 65, 68–70], or maintained contact with family or friends in the country of origin (*n* = 16, 34.0%) [13, 21, 27, 36, 38, 39, 42–45, 52–54, 65, 68, 69]. A number of studies (*n* = 9, 19.1%) inquired as to whether migrants had either a confidant (e.g. a person they could rely on or confide in) or a partner in the country of origin [33, 37–39, 41, 45, 54, 58, 70]. Authors often measured the number of visits or contacts made since arrival in the destination country or inquired as to how frequently contacts/visits were made. The studies on transnational families (*n* = 11, 23.4%) most often only assessed whether migrants had family members or children in another country [37, 50, 55–57, 59–63, 67]. Three studies (6.4%) measured transnational parents’ frequency of contact with their children [57, 63, 67], and one of these (2.1%) also examined whether remittances were sent monthly [67].

Remittance sending was measured in all but two of the studies that included economic transnational ties (*n* = 24, 51.0%) [13, 21, 22, 27–30, 32–34, 38, 40, 41, 45, 46, 51–53, 58, 64, 65, 67–69]. Most often (*n* = 15, 31.9%), migrants were asked whether they had sent remittances since arriving in the destination country [13, 22, 28, 30, 33, 34, 38, 40, 41, 46, 51, 52, 65, 68, 69]; three studies (6.4%) asked whether migrants had remitted in the last year [21, 27, 53], and five (10.6%) asked about frequency of remittances [32, 45, 58, 64, 67]. Six studies (12.8%) examined remittance burden, as measured by the average amount and/or the percentage of income sent [29, 30, 40, 46, 51, 64]. One study, which focused on migrant nurses, asked respondents to indicate who they were supporting [51]. Eleven studies (23.4%) examined other types of economic ties including business transactions (e.g. being an owner or an investor in a business in the country of origin, or visiting for business purposes) [13, 21, 27, 45, 62, 68, 69]; financial assets (e.g. owning land or property) [13, 21, 27, 32, 53, 59, 62, 68, 69]; purchasing items from their country of origin [13, 58, 69]; and sending donations/funding for projects (e.g., to build a school or healthcare centre) [21, 27, 68].

Studies assessing transnational attitudes and identity most often inquired about migrants’ return intentions (*n* = 7, 14.9%) [32, 33, 36, 45, 58, 66, 70]. A few studies asked about feelings of loss or attachment to their country of origin (*n* = 4, 8.5%) [36, 38, 39, 45], or whether migrants identified with their country’s heritage (*n* = 2, 4.3%) [47, 68]; one study (2.1%) asked about citizenship in the home country [34].

Cultural ties were examined by asking migrants about their participation in cultural activities (*n* = 8, 17.0%) [13, 21, 27, 30, 39, 45, 68, 69], or their media consumption, such as reading newspapers (*n* = 6, 12.8%) [21, 27, 38, 39, 53, 68] or watching TV, using the internet and listening to the radio (*n* = 5, 10.6%) [21, 27, 39, 53, 58] from their country of origin. A few studies inquired about migrants’ religiosity (*n* = 4, 8.5%) [13, 33, 39, 69] and language use (*n* = 3, 6.4%) [38, 39, 52].

Political ties were primarily assessed by asking migrants about their interest in the politics of their country of origin (*n* = 6, 12.8%) [13, 21, 27, 39, 68, 69]. Participation in associations (*n* = 4, 8.5%) [13, 32, 68, 69], voting in their home country’s elections (*n* = 1, 2.1%) [32], giving monetary donations to political candidates (*n* = 2, 4.3%) [13, 32], and participation in political demonstrations (*n* = 1, 2.1%) [68], were also assessed.

Among the studies measuring transnational healthcare use, two (4.3%) examined migrants’ use of different types of health services (e.g. physician, specialist, inpatient care, purchasing/receiving medication from their country of origin) [48, 49], while one study respectively (2.1%), focused on transnational dental care [31], and visits to a physician in the country of origin [35]. Three studies (6.4%) assessed the timing of transnational healthcare use (e.g. frequency of use or use since arrival in destination country), but did not assess the type of care sought [36, 47, 54].

### Health and well-being outcomes studied

A summary of the examined outcomes in relation to transnationalism is presented in Table 3. Almost half (49%) of the research focused on mental health outcomes [13, 22, 29, 30, 34, 36, 37, 39, 44–46, 50, 52, 55–57, 59–64, 69] and 38% (*n* = 18) examined general health or well-being [13, 21, 31, 35, 38, 40–42, 45, 47, 48, 52, 55–59, 62]. Socioeconomic indicators, social contacts and relationships, and integration/identity outcomes were studied in 30% (*n* = 14) [31, 34, 35, 40, 47–49, 51–54, 60, 64, 66], 19% (*n* = 9) [13, 33, 48, 53–55, 66–68] and 23% (*n* = 11) [13, 32, 34, 35, 47, 51, 65, 66, 68–70] of studies, respectively. Health behaviours (*n* = 4, 9%) [27, 28, 43, 60] and employment related outcomes (*n* = 2, 4%) [52, 63] were examined less often. Many studies showed mixed associations because different dimensions of transnationalism (e.g., social vs. economic ties) or of the outcomes (e.g., fertility behaviour vs. ideals) were examined, and/or because results varied for different populations (e.g., men/women, migrants from different regions or with varying lengths of time in the host country or different generations). Some studies also used a variety of ways to operationalize a specific dimension of transnationalism (e.g., remittance sending as well as the amount of remittances) and/or an outcome (e.g., *general* fertility ideals vs. *personal* fertility ideals).

**Table 3:** Health and well-being outcomes studied

Outcomes studied	n^a^	%^a^	Studies
Mental health	23	48.9%	
Major depressive episodes or symptoms	9	19.1%	[13, 29, 34, 44, 46, 52, 55, 60, 61]
Poor emotional well-being	5	10.6%	[30, 45, 56, 59, 62]
Loneliness	2	4.3%	[36, 50]
Acculturative stress	2	4.3%	[60, 69]
Anxiety	2	4.3%	[13, 37]
Psychological distress	2	4.3%	[22, 39]
Happiness	4	8.5%	[39, 57, 59, 63]
Sadness	1	2.1%	[46]
Poor mental health	2	4.3%	[34, 57]
Immigration related stress	1	2.1%	[60]
Marital stress	1	2.1%	[60]
Employment stress	1	2.1%	[60]
General stress	1	2.1%	[13]
Emotional financial strain	1	2.1%	[64]
Partner violence	1	2.1%	[60]
Health & well-being	18	38.3%	
Poor self-rated health	6	12.8%	[21, 35, 41, 56, 57, 59]
Life satisfaction	7	14.9%	[13, 45, 55–57, 59, 62]
Poor subjective well-being	1	2.1%	[58]
Dental problems	1	2.1%	[31]
Chronic disease & health limitations	2	4.3%	[21, 35]
Health satisfaction	1	2.1%	[38]
Inflammatory markers	1	2.1%	[42]
General health problems	5	8.5%	[40, 47, 48, 52, 62]
Socioeconomic indicators	14	29.8%	
Financial strain	1	2.1%	[64]
Housing	3	4.3%	[40, 51, 52]
Low income	4	6.4%	[31, 35, 53, 60]
High income	2	4.3%	[35, 52]
Financial struggles	1	2.1%	[51]
Socioeconomic position	1	2.1%	[48]
Satisfaction with host country economy	1	2.1%	[34]
Knowledge, positive perception and/or use of community/social resources	2	4.3%	[52, 66]
Lack of health/dental insurance	6	8.5%	[31, 47, 49, 52–54]
Poor quality healthcare in host country	3	2.1%	[47, 49, 53]
Has a usual care provider	1	2.1%	[47]
Social contacts & relationships	9	19.1%	
Quality of parent-child relationships (in host country)	1	2.1%	[55]
Quality of parent-child relationships (children abroad)	1	2.1%	[67]
Social support	1	2.1%	[13]
Social networks	1	2.1%	[33]
Relationships with locals	2	4.3%	[66, 68]
Transnational relationships	3	2.1%	[48, 53, 54]
Health Behaviours	4	8.5%	
Alcohol use	1	2.1%	[43]
Smoking	1	2.1%	[28]
Substance abuse	1	2.1%	[60]
Fertility ideals, current/cumulative fertility	1	2.1%	[27]
Integration	11	23.4%	
Perceived discrimination	6	12.8%	[13, 32, 35, 66, 69, 70]
Citizenship in host country	1	2.1%	[47]
Integrated in host country	1	2.1%	[35]
Knowledge of host country’s language	2	4.3%	[47, 66]
Sense of belonging in host country	1	2.1%	[66]
Host country identity	2	4.3^%^	[65, 68]
Happy with decision to have moved to host country	1	2.1%	[34]
Use of country of origin as reference group to evaluate social standing	1	2.1%	[34]
Intention to leave host country	1	2.1%	[51]
Home country identity	1	2.1%	[65]
‘Ethnic group’ identity	2	4.3%	[13, 65]
Employment related outcomes	2	4.3%	
Job absenteeism	1	2.1%	[63]
Job instability	1	2.1%	[63]
Work-family life conflict	1	2.1%	[63]
Unemployment	1	2.1%	[52]

^a^Numbers sum up to greater than 47 and 100% since a number of studies examined more than one outcome

Social transnational ties (visits to the home country, long-distance communication) were shown to be associated with several positive outcomes including perceived social support [13], a stronger ethnic identity [13], general well-being [45], life satisfaction [45], good self-rated health [41], reduced levels of anxiety [13], stress [13], and depression (among men) [44], and lower levels of inflammatory markers [42]. However, quite a few studies also showed associations with negative outcomes including depression among women [29, 44], poorer well-being [58], psychological distress [39] and decreased emotional well-being [45]. A number of studies also reported transnational parenting as being associated with negative outcomes including lower life satisfaction [57, 59], worse emotional well-being [59], less happiness [57, 59], poorer health status [57, 59], having a chronic condition among women [21], depression among mothers [61], and a higher likelihood of experiencing job instability and family-to-work conflict [63]. Other studies however, noted that associations between transnational parenthood and poor outcomes (lower life satisfaction, poorer health status, worse emotional well-being, anxiety and depressive symptoms) disappeared when socio-demographic factors, such as educational and wealth status, documentation status, and length of stay in destination country, were controlled [37, 56, 62].

Remittance sending also showed a mix of results. Remitting was associated with lower odds of smoking [28], improved emotional wellness among women over time [30], less emotional strain [64] and lower levels of depression [29], but also with housing issues [40, 52], poorer subjective well-being [45, 58], depression [46] and sadness [46].

Regarding cultural ties, media consumption was associated with lower levels of happiness among first generation migrants in one study [39], but generally participation in cultural activities and media consumption appeared to not really have an influence on health and well-being [39, 45, 58]. However, in some studies cultural and social ties were examined together [13, 39, 68, 69], so it is difficult to draw conclusions. Similarly, political ties, also tended to not be examined separately from other ties [13, 21, 27, 32, 39, 69], so clear results could not be determined. Regarding attachment to one’s home country, as measured by intentions to return, positive associations were shown with poorer general well-being [58], having a chronic condition among men [21], perceived discrimination [66], and a lesser sense of belonging in the host country [66]. Similarly, thinking about moving back was negatively associated with emotional well-being and life satisfaction [45]. Feelings of loss with regards to one’s home country were also shown to be associated with more loneliness [36].

Lastly, transnational health/dental care utilization was associated with various factors including having health or dental problems [31, 35], a lack of insurance [31, 47] and experiences of discrimination or poor quality healthcare in the host country [35, 47]. Migrants who maintained social ties with relatives back home were also more likely to use transnational health services [54]. There were conflicting results between studies regarding the association between integration and transnational healthcare use, where De Jesus and Xiao (2013) [47] found that having citizenship or permanent residency in the destination country was positively associated with seeking transnational healthcare, but Kemppainen et al. (2018) [35] found that with greater integration in the destination country, migrants were less likely to seek transnational health services. De Jesus and Xiao (2013) [47] explained their results with the reasoning that the risk for undocumented migrants to cross the border for health care may be too high, whereas Kemppainen and colleagues (2018) [35] included citizenship within a variable that encompassed several other components of integration (i.e. length of stay in destination country, citizenship status, subjective nationality, the amount of friends from the destination country, and proficiency of the host country’s language).

### Methodological quality of studies

Among the quantitative studies, the majority (*n* = 28) applied an adequate sampling strategy and had a representative sample [21, 22, 27–50, 65, 70]; 15 studies employed non-random approaches for recruitment such as convenience, or snowball sampling [13, 55–64, 66–69]. Across the studies, the measures used to examine the health and well-being outcomes were mostly known standardized, validated tools, whereas for the tools/questions measuring transnationalism there tended to be little to no validity and reliability information provided (see below). In all but two studies [13, 60], researchers controlled for potential confounders including gender/sex and socioeconomic and migration factors. The studies were mainly cross-sectional, so often the direction of the relationships between transnationalism and health and well-being outcomes could not be confirmed.

For all of the mixed-methods’ studies [51–54], the design and the mixing of the qualitative and quantitative portions were appropriate. For the qualitative portions of the studies, the methods used were suitable and the results were adequately supported by the data. In one study, participants were recruited through convenience/purposive sampling, and the qualitative and quantitative data collection occurred concurrently [52]. In another study, the participants for the quantitative survey were based on purposive, non-random sampling and in-depth interviews were then held subsequently; interview participants included some of the participants from the quantitative portion [54]. The third study involved a large Canadian-wide survey and focus groups with participants who were recruited from one metropolitan city through snowball sampling; the survey data were used to describe the population, while the focus group participants completed a questionnaire on transnational behaviour and health to complement the qualitative data collected on health and experiences of transnational healthcare use [53]. In the fourth study, a random sample was selected to complete a survey that was informed from the qualitative portion of the study [51]. Tools used to measure health/well-being outcomes were well described in two studies [52, 54]; in one study there was little information about the questions used to measure well-being [51]; and in the other study a validated survey was used to generally describe the health outcomes of the population, but no information was provided about the tool used to measure the health of the focus group participants [53]. The analyses for the quantitative portions for all four studies were descriptive.

### Validity and reliability of the transnationalism measures

Overall, very few studies provided reliability or validity information. Seven studies (14.9%) reported internal consistency using Chronbach’s alpha (C). Two studies (4.3%) had poor internal consistency (C < 0.70) [21, 27], and five studies (10.6%) had moderate to high internal consistency (C = 0.70–0.87) although not for all dimensions [13, 36, 39, 68, 69]; the number of items ranged from 9 to 21 and samples were from just over one hundred to more than 2000 (see Table 2 and Appendix 1). Validity was rarely mentioned, and when discussed, was largely based on the tools having been developed and informed from existing literature on transnationalism. In some studies, the analyses were based on existing datasets and transnationalism was not a primary focus in the original data collection, so the validity of the transnationalism measures is not clear [30, 31, 35, 37, 40, 46, 47, 70]. For a few studies the measures were informed by the researchers own earlier work with migrants [21, 27, 38, 45]. Afulani et al. (2015) performed a principal component analysis and the same scale was used in another study by the same author [27]. Similarly, Murphy et al. (2004) performed a factor analysis [13], and two subsequent studies conducted by different researchers used this initial work as the basis for creating their transnationalism measures [39, 69]. Two studies worked closely with migrant communities to inform the development of their data collection tools [48, 64]. A handful of studies provided detailed information about their transnationalism measures, including the theoretical basis for their development [13, 21, 27, 38, 39, 45, 65, 68].

## Discussion

We identified 47 studies that measured transnationalism and examined health and well-being in migrants in high-income countries. Approximately half of the studies took place in the United States and just over 20% were conducted in the Netherlands; study samples most often consisted of migrants originating from Latin America, sub-Saharan Africa, East/Southeast Asia and/or Eastern Europe. The majority of studies focused on social or economic ties between immigrants, broadly defined, and their countries of origin. Almost half of the studies examined mental health outcomes, such as emotional well-being, or symptoms of major depression, followed by self-rated health, life satisfaction and perceived discrimination.

Transnationalism was not explicitly or well-defined in a number of studies, although in some instances this was due to the fact that the study focused on a specific dimension of transnationalism. Definitions, whether implied or explicit, most commonly focused on social connections between migrants and their countries of origin, or included other types of interactions across borders that require maintenance of social ties, such as remittance sending. In the social sciences literature there is an extensive amount of theorizing and writing on the concept of transnationalism and definitions reflect the complex, dynamic and processual nature of this phenomenon [3]; for example Schiller et al. (1992) described transnationalism as “the processes by which immigrants build social fields that link together their country of origin and their country of settlement” which include a range of overlapping activities, relationships and social networks that are continuously evolving and shifting, and that are intentional, meaningful, and that contribute to dual or plural identities and belongingness [4]. And through the various activities and relationships, there is movement of ideas, resources and information. Transnationalism has also been described as a transformative process that entails the creation of new social spaces [3, 71]. As noted by others, there is no universal definition of transnationalism, and for pragmatic purposes, elaborate, theorized definitions have limited use in empirical quantitative research as its challenging to delineate clear variables [3]. The conceptualization of transnationalism is thus usually narrower and often determined by the purpose and scope of the study. This proved to be the case for many of the studies identified in this review.

The measures of transnationalism identified in the review mostly assessed for social ties such as contact with family/children and friends, and remittance sending. Studies and measures also tended to focus on only one or two dimensions of transnationalism, rather than examine several forms of transnational ties. In the social sciences, social and economic dimensions of transnationalism are also frequently measured, however, there also appears to be more consideration for cultural, attitude/identity, and political dimensions [72–77]. According to Tedeschi et al. (2020), who conducted a review of the concept and current debates regarding transnationalism in the most recent and cited literature, key categories or aspects that have been measured include sociocultural, political and economic activities, and sense of belonging [3]. Although there is considerable overlap between transnational social and cultural ties since the latter are often maintained via connections with family and friends, the important influence that culture can have on how one perceives and experiences health, and how one chooses to respond and treat illnesses, greater attention to transnational cultural ties in health research, is warranted. Similarly, transnational identity/sense of belonging merit more consideration since the results from our review suggest that these dimensions can be associated with varying levels of loneliness and emotional well-being [36, 45].

Two other key points raised by Tedeschi et al. (2020) in their review vis à vis the dimensions to measure, were, one, the lack of research on transnational healthcare use, and two, the debate on how to consider integration (in the destination country) in relation to the concept of transnationalism [3]. The results of our review corroborate the point on the need for more research on the use of transnational healthcare, as we found only a handful of studies that measured this dimension, and healthcare use is an important determinant of health and well-being. Regarding integration, this was mostly deemed an outcome in our review, as it is a social indicator of well-being due to the social benefits and rights that can come with being integrated into a host-society and since it was shown to be influenced by (or influence) other dimensions of transnationalism such as transnational healthcare use [35, 47] and intentions to return to one’s home country [66]. This is consistent with the idea that integration is a distinct concept that can either be reinforced or diminished through transnational ties or vice versa. However, integration can also be considered an aspect of transnationalism as it can be considered an indicator of dual/plural identity and belonging [74]. Whatever the case, it underscores the importance that integration is a key variable to contemplate in migrant health studies, whether it be as an outcome, a complementary variable, or as an element of transnationalism, as it allows for a better understanding of the complexity of migrants’ lives. It also highlights the need for studies to have clarity regarding the conceptualization of transnationalism, even if narrowly focused.

The review also highlights issues regarding the operationalization of transnationalism. Generally, transnationalism was not operationalized consistently across the research, including the formulation, number and method of combining items used to represent the particular dimensions of transnationalism. For instance, some authors focused on participation in cultural activities, whereas others examined consumption of media from their country of origin, in order to measure cultural ties. There were only a few items that were used across a number of studies (i.e., whether migrants had remitted or made a return visit or had contact with family and friends since arrival, or in the past year; and number of remittances or visits). Moreover, in some studies, dimensions were combined to generate ‘transnationalism’ scores whereas in other studies, dimensions were maintained as separate variables and in a dichotomous form. Evaluating the quality of the measures was difficult because reliability and validity data, including information on the cultural appropriateness and the language translation procedures, were not adequately reported. The multiple ways transnationalism was operationalized gave way to challenges in the comparability, generalizability, and transferability of results.

Inconsistencies in the selection and operationalization of variables and the use of diverse methods to capture transnationalism, are issues in the social sciences as well [3]. Although context will vary across studies and so universal measures are not really attainable or desirable [3], researchers should draw on existing and validated items/questions in order to optimize comparability across research; in that regard, this review can be a useful resource as it provides a pool of studies to draw from to inform future work. The summary of the operationalization of the transnationalism measures reported in Appendix II (Additional file 2) and the validity and reliability information reported in the results section above, as well as the list of additional references provided in Appendix I, column 3 (Additional file 2), which provide supplementary information on data/sample sources or the parent projects for a number of the studies, can be especially helpful for assisting researchers in selecting the best questions and instruments for measuring transnationalism in their research.

Furthermore, the results of the review reveal that different types of transnational ties can have diverse impacts on health and well-being, and thus suggest that using one dichotomous variable (transnational vs. non-transnational) or a crude score to represent all dimensions of transnationalism together, is an overly reductionist approach. Results also show that the relationships between transnational ties and health and well-being are complex and that using multifaceted measures for each distinct dimension, or for when there is some overlap between the dimensions (e.g., social and cultural, or social and economic ties), is the best approach. For example, when measuring social transnational ties, it seems imperative to take into account the quality and frequency of contacts as well as the nature of family separation (e.g., whether it is forced or not and whether visits are possible). Similarly, for remittance sending, the level of financial burden as well as who or what the remittances are for (children, or other family members; health or education), and whether or not one feels they are meeting their economic obligations, are important to assess. Socio-demographics, including gender and migration factors, are also essential variables to include as they may confound or mediate effects. Similar recommendations have been made previously, Vertovec (2003) for example, suggested that for transnational social ties, the frequency, intensity, regularity, and who (person, institution, place) should be incorporated into the measurement [71]. Meaning of the relationships and motivations underpinning the maintenance of transnational connections (e.g., as a sense of duty, as an outlet to compensate for social standing) have also been recommended by others [3, 78]. To reflect this level of complexity, methods such as latent variable modelling, as done by Burholt et al. (2016) to examine transnational family relationships [65], and by Ciobanu et al. (2020), who used multiple indicators including remittance sending, family ties and nationality, to create classes of transnationalism [72], are effective. These methods, however, may not always be feasible due to the need for larger sample sizes. Generating scores using items that capture the same aspect of a transnationalism dimension (e.g., missing home, having a sense of loss, and intends to return) and/or combining various items to create categories (e.g., remittance sending and experiences financial burden vs. remittance sending and *not* experiencing financial burden) can also be informative approaches for operationalizing transnationalism.

Qualitative research suggests that there are other aspects of transnationalism that may warrant being studied quantitatively. One such aspect is transnational caregiving, a form of transnationalism where a migrant provides emotional or financial support, or arranges care for elderly and ailing parents across borders [79, 80]. Transnational caregiving has been described in resulting in feelings of distress, and a loss of control in migrants, and yet we found no quantitative studies measuring transnational caregiving in relation to health and well-being. ‘Transnational fostering’, whereby children are sent abroad to live with relatives for short or extended periods of time, either for the purpose of childcare or for socializing and disciplining children, is another form of transnationalism that has also been identified in the qualitative literature [81, 82]. These transnational ties may have implications for the health and well-being of migrant parents, for example as a source of social support, and/or for children, for example, as a determinant shaping their socialization and development. In the context of health, it would also be worthwhile to have more developed measures of transnational health services’ use, including informal sources of medical advice and support. Qualitative studies have suggested that family and social networks may be sources of medical information and traditional medicines and therapies, particularly for managing chronic illness and during pregnancy and the postpartum period [83–85]. Overall, more work is needed to further develop and validate tools for measuring transnationalism specifically for health research.

The studies in our review examined a variety of outcomes related to health and well-being, and generally their conclusions had similarities to the qualitative studies. Overall, the narratives of migrants indicate both negative mental health and well-being associated with maintaining transnational ties, such as, feelings of guilt, loss or up-rootedness, and being over-burdened, as well as positive outcomes, like increased self-esteem, and a sense of belonging that acts as a resource to cope with challenges faced in the host country [19, 86–88]. However, the quantitative research done thus far has mainly been cross-sectional making it difficult to draw conclusions regarding the temporal sequence of the relationships. It is plausible that mental and physical health, as well as social factors, can lead to more or less transnationalism including transnational practices, exchanges and identities. Longitudinal research designs would therefore help elucidate whether health and well-being indicators are predictors and/or outcomes and also bring to light variations in relationships over time. In addition, qualitative studies have also shown that transnationalism affects health behaviours and beliefs, such as dietary behaviors, management of hypertension, and chronic health beliefs, and quantitative studies on such outcomes were sparse in the review. This suggests a need for future studies to investigate how transnationalism may impact migrants’ ways of managing illnesses and behaviors that protect or place them at risk for diseases [12, 89–92].

The review also highlights a need for more research with different populations. Studies examining migrants with health disorders or infirmities were scant, making it difficult to know whether the tools and findings are transferable to less healthy migrants. Migrants were generally grouped all together and refugees and asylum-seekers were less represented in the research. The transnational network and experiences may look very differently for refugees and asylum-seekers due to the difficult and/or precarious nature of their migration. Migration history and status are also known to affect health, for example mental health, pregnancy outcomes, and the health of children, as well as access to services in the destination country [93, 94]. Thus it would be important for future studies to determine whether transnationalism affects more vulnerable migrants similarly or differently compared to economic and family sponsored migrants. Moreover, although studies examining the health and well-being outcomes of children and family left-behind exist, we found no literature that simultaneously examined the impact of transnationalism on health and well-being of migrants and their family back home [95–97].

An extensive systematic search strategy was undertaken and so we can assume that most of the studies relevant to transnationalism and health and well-being were included. However, our search strategy may not have identified those studies where migrants’ ties were not explicitly described as cross-border or transnational especially since we did not use search terms that would have identified studies examining specific dimensions of transnationalism. We also excluded studies whose sole focus was language maintenance or cultural practices, although transnationalism encompasses these facets, and these studies may also have shed light on transnationalism and health and well-being. We only provided a general overview on the relationships between transnationalism and health and well-being and we did not report on how the outcomes were defined and measured, which likely explains some of the inconsistent results found. This review therefore does not allow for definitive conclusions to be drawn regarding the associations between transnationalism and the health and well-being outcomes. Despite these limitations, this scoping review provides a comprehensive overview of the ways transnationalism has been defined and quantitatively studied in relation to migrants’ health and well-being in high-income countries and offers a number of points for consideration for future work.

## Conclusion

Transnationalism is a multi-dimensional, complex concept that is increasingly recognized as impacting migrants’ health and well-being in high-income countries. To enhance comparability in this field of research, researchers should provide a clear, explicit definition of transnationalism (and/or of its dimensions) based on the scope of their study, and for its measurement, they should draw from validated items/questions and be consistent in its operationalization across studies. To enhance the quality of findings, reductionist approaches for operationalizing transnationalism, such as a crude overall score or a dichotomous (transnational vs. non-transnational) variable, should be avoided; more complex approaches (e.g., latent variable modelling) should be employed. Use of longitudinal designs would also improve the interpretability of the temporal sequence of association between transnationalism and health and well-being outcomes. Further research on other transnational ties, beyond social contacts with family and friends back home and remittance sending, and with a diversity of migrant populations, and on other health and well-being outcomes (other than mental health), is warranted.

## Supplementary Information

**Additional file 1.** Search strategies by database.

**Additional file 2.** Appendices. Appendix I: Summary of literature & Appendix II: Summary of the operationalization of the transnationalism measures.

## Data Availability

All information and data reported in this review are retrievable from the original sources.
