# Supplementary material for: A scoping review on the measurement of transnationalism in migrant health research in high-income countries
Source: Global Health. 2021 Oct 29;17:126. doi: 10.1186/s12992-021-00777-2 (PMC8555176; doi:10.1186/s12992-021-00777-2)
Supplement: Supplementary file 1 — Additional file 1. Search strategies by database. [file 12992_2021_777_MOESM1_ESM.docx]

Additional File 1 – Search strategies by database

Table 1. MEDLINE

| Search Step | Combination of search terms | Search Field |
| --- | --- | --- |
| Main search area – Population | | |
| #1 | Transnational* OR transmigrant* OR cross-border ties | Title, abstract, & keywords |
| #2 | immigra* OR migra* OR emigra* OR refugee* OR asylum seeker* | Title, abstract, & keywords |
| #3 | transients and migrants OR emigrants and immigrants OR refugees | MeSH Terms |
| #4 | #1 OR #2 OR #3 | |
| Main search area – Health, well-being, & social support | | |
| #5 | Health* OR mental health OR psychological distress OR stress* OR well#being OR quality of life OR social support OR social isolation OR social network* OR loneliness OR intergenerational relations | Title, abstract, & keywords |
| #6 | Health OR Health Behavior OR Attitudes to Health OR Mental Health OR Stress, Psychological OR Psychological distress OR Quality of Life OR Social support OR Social isolation OR Loneliness OR intergenerational relations | MeSH Terms |
| #7 | « Social ties » adj5 (Health* OR mental health OR psychological distress OR stress* OR well#being OR quality of life OR social support OR social isolation OR social network* OR loneliness OR intergenerational relations) | All fields |
| #8 | #5 OR #6 |  |
| #9 | (#1 AND #8) OR (#4 AND #7) |  |
| #10 | #9 NOT (infectious disease* OR epidemiolog* OR corporation OR tobacco industry) |  |
|  | Limit #10 to English or French |  |

Table 2. Global Health

| Search Step | Combination of search terms | Search Field |
| --- | --- | --- |
| Main search area – Population | | |
| #1 | Transnational* OR transmigrant* OR cross-border ties | Title, abstract, & heading word |
| #2 | immigra* OR migra* OR emigra* OR refugee* OR asylum seeker* | Title, abstract, & heading word |
| #3 | migrants or immigrants or refugees | Subject heading |
| #4 | #1 OR #2 OR #3 | |
| Main search area – Health, well-being, & social support | | |
| #5 | health* or stress* or well#being or quality of life or social support or social isolation or social network* or loneliness | Title, abstract, & heading word |
| #6 | Health or health indicators or health care or health behavior or health beliefs or health care utilization or health inequalities or mental health or stress or "quality of life" or support systems or social interaction or social participation or loneliness | Subject Heading |
| #7 | « Social ties » adj5 (Health* OR mental health OR stress* OR well#being OR quality of life OR social support OR social isolation OR social network* OR loneliness OR intergenerational relations) | All fields |
| #8 | #5 OR #6 |  |
| #9 | (#1 AND #8) OR (#4 AND #7) |  |
| #10 | #9 NOT (infectious disease* OR epidemiolog* OR corporation OR tobacco industry) |  |
|  | Limit #10 to English or French |  |

Table 3. PsycINFO

| Search Step | Combination of search terms | Search Field |
| --- | --- | --- |
| Main search area – Population | | |
| #1 | Transnational* OR transmigrant* OR cross-border ties | Title, abstract, & key concepts |
| #2 | immigra* OR migra* OR emigra* OR refugee* OR asylum seeker* | Title, abstract, & key concepts |
| #3 | exp Human Migration or exp Immigration or exp Refugees or exp Asylum Seeking | Subject heading |
| #4 | #1 OR #2 OR #3 | |
| Main search area – Health, well-being, & social support | | |
| #5 | Health* or mental health or stress* or well#being or quality of life or social support or social isolation or social network or loneliness | Title, abstract, & key concepts |
| #6 | exp "Health Related Quality of Life" or exp Health Status or exp Health Care Utilization or exp Health or exp Health Attitudes or exp Mental Health or exp Health Behavior or exp Stress or Interpersonal Relationships or Social Networks or Loneliness | Subject Heading |
| #7 | « Social ties » adj5 (health* or mental health or psychological distress or stress* or well#being or quality of life or social support or social isolation or social network* or loneliness) | All fields |
| #8 | #5 OR #6 |  |
| #9 | (#1 AND #8) OR (#4 AND #7) |  |
| #10 | #9 NOT (infectious disease* OR epidemiolog* OR corporation OR tobacco industry) |  |
|  | Limit #10 to English or French |  |

Table 4. Embase

| Search Step | Combination of search terms | Search Field |
| --- | --- | --- |
| Main search area – Population | | |
| #1 | Transnational* OR transmigrant* OR cross-border ties | Title, abstract, & keywords |
| #2 | immigra* OR migra* OR emigra* OR refugee* OR asylum seeker* | Title, abstract, & keywords |
| #3 | Migrant or migrant worker or undocumented immigrant or immigrant or refugee or migration or asylum seeker | Subject heading |
| #4 | #1 OR #2 OR #3 | |
| Main search area – Health, well-being, & social support | | |
| #5 | Health* or stress* or psychological distress or well#being or quality of life or social support or social isolation or social network* or loneliness | Title, abstract, & keywords |
| #6 | Attitude to health or health care utilization or health behavior or exp stress or stress management or parental stress or quality of life or human relations or social support or social isolation or social network or loneliness | Subject Heading |
| #7 | « Social ties » adj5 (health* or mental health or psychological distress or stress* or well#being or quality of life or social support or social isolation or social network* or loneliness) | All fields |
| #8 | #5 OR #6 |  |
| #9 | (#1 AND #8) OR (#4 AND #7) |  |
| #10 | #9 NOT (infectious disease* OR epidemiolog* OR corporation OR tobacco industry) |  |
|  | Limit #10 to English or French |  |

Table 5. CINAHL

| Search Step | Combination of search terms | Search Field |
| --- | --- | --- |
| Main search area – Population | | |
| #1 | Transnational* OR transmigrant* OR cross-border ties | Title, & abstract |
| #2 | immigra* OR migra* OR emigra* OR refugee* OR asylum seeker* | Title, & abstract |
| #3 | Transients and Migrants OR Immigrants, Illegal OR Immigrants OR Emigration and Immigration OR Refugees | Subject heading |
| #4 | #1 OR #2 OR #3 | |
| Main search area – Health, well-being, & social support | | |
| #5 | Health* OR stress* OR well#being OR quality of life OR social support OR social isolation OR social network* OR loneliness | Title, & abstract |
| #6 | Wellness OR Attitude to Health OR Health Behavior OR Mental Health OR Psychological Well-Being OR Quality of Life OR Stress OR Stress, Psychological OR Health OR Support, Psychosocial OR Social Isolation OR Social Networks OR Loneliness | Subject Heading |
| #7 | « Social ties » N5 (health* OR mental health OR psychological distress OR stress* OR well#being OR quality of life OR social support OR social isolation OR social network* OR loneliness) | All fields |
| #8 | #5 OR #6 |  |
| #9 | (#1 AND #8) OR (#4 AND #7) |  |
| #10 | #9 NOT (infectious disease* OR epidemiolog* OR corporation OR tobacco industry) |  |
|  | Limit #10 to English or French |  |

Table 6. Anthropology Plus

| Search Step | Combination of search terms | Search Field |
| --- | --- | --- |
| Main search area – Population | | |
| #1 | Transnational* OR transmigrant* OR cross-border ties | Title, & subject heading |
| #2 | immigra* OR migra* OR emigra* OR refugee* OR asylum seeker* | Title, & subject heading |
| #3 | #1 OR #2 |  |
| Main search area – Health, well-being, & social support | | |
| #4 | Health* OR stress* OR well#being OR quality of life OR OR psychological distress OR interpersonal relations OR social support OR social isolation OR social network* OR loneliness | Title, & subject heading |
| #5 | « Social ties » adj5 (health* OR mental health OR psychological distress OR stress* OR well#being OR quality of life OR social support OR social isolation OR social network* OR loneliness) | All fields |
| #6 | (#1 AND #4) OR (#3 AND #5) |  |
| #7 | #6 NOT (infectious disease* OR epidemiolog* OR corporation OR tobacco industry) |  |
|  | Limit #10 to English or French |  |

Table 7. Sociological Abstracts

| Search Step | Combination of search terms | Search Field |
| --- | --- | --- |
| Main search area – Population | | |
| #1 | Transnational* OR transmigrant* OR cross-border ties | Title, abstract, keyword, & main subject |
| #2 | immigra* OR migra* OR emigra* OR refugee* OR asylum seeker* | Title, abstract, keyword, & main subject |
| #3 | #1 OR #2 |  |
| Main search area – Health, well-being, & social support | | |
| #4 | Health* OR stress* OR well#being OR “quality of life” OR “mental health” OR “psychological distress” OR “interpersonal relations” OR “social support” OR “social isolation” OR “social network*” OR loneliness | Title, abstract, keyword, & main subject |
| #5 | “social ties” NEAR/5 (health* OR stress* OR well#being OR “mental health” OR “psychological distress” OR “social support” OR “social isolation” OR “social networks” OR loneliness) | All fields |
| #6 | (#1 AND #4) OR (#3 AND #5) |  |
| #7 | #6 NOT (infectious disease* OR epidemiolog* OR corporation OR “tobacco industry” OR “rural urban migration” OR “internal migration”) |  |
|  | Limit #10 to English or French |  |

Table 8. ProQuest Central

| Search Step | Combination of search terms | Search Field |
| --- | --- | --- |
| Main search area – Population | | |
| #1 | Transnationalism or “transnational ties” or “cross-border ties” or transmigrant* | Title, & subject heading |
| Main search area – Health, well-being, & social support | | |
| #2 | Health* OR stress* OR well#being OR quality of life OR OR psychological distress OR interpersonal relations OR social support OR social isolation OR social network* OR loneliness | Title, & subject heading |
| #3 | (#1 AND #2) |  |
| #4 | #3 NOT (infectious disease* OR epidemiolog* OR corporation OR “tobacco industry” OR “rural urban migration” OR “internal migration”) |  |
|  | Limit #10 to English or French |  |

Table 9. Web of Science

| Search Step | Combination of search terms | Search Field |
| --- | --- | --- |
| Main search area – Population | | |
| #1 | Transnationalism or “transnational ties” or “cross-border ties” or transmigrant* | Topic, & title |
| Main search area – Health, well-being, & social support | | |
| #2 | Health* OR stress* OR well#being OR quality of life OR psychological distress OR interpersonal relations OR social support OR social isolation OR social network* OR loneliness | Topic, & title |
| #3 | (#1 AND #2) |  |
| #4 | #3 NOT (infectious disease* OR epidemiolog* OR corporation OR “tobacco industry” OR “rural urban migration” OR “internal migration”) |  |
|  | Limit #10 to English or French |  |
