# Supplementary material for: A scoping review on the measurement of transnationalism in migrant health research in high-income countries
Source: Global Health. 2021 Oct 29;17:126. doi: 10.1186/s12992-021-00777-2 (PMC8555176; doi:10.1186/s12992-021-00777-2)
Supplement: Supplementary file 2 — Additional file 2. Appendices. Appendix I: Summary of literature & Appendix II: Summary of the operationalization of the transnationalism measures. [file 12992_2021_777_MOESM2_ESM.docx]

**Appendix I: Summary of Literature**

| 1^st^ author, Year  (Reference #) | Study objectives | Type of research | Location  &  Data/sample source or parent project ^+^ | Method of data collection | Population ^++^  &  Length of time in destination country (L) | Language of data collection | Sample size | General Findings |  |
| --- | --- | --- | --- | --- | --- | --- | --- | --- | --- |
| Afulani, 2015 (1) | To examine the effects of socialization, adaptation, and transnationalism on the fertility behavior and fertility ideas of sub-Saharan African migrants in France | Survey, cross-sectional | France  Trajectoires et Origines survey (2010)^1^ | In-person interview | Sub-Saharan Africans and non-Sub-Saharan African migrants ages 18-50 (NM, IM,IM2)  L: <5 to 25 years (IM) | French,  Unspecified mother tongue | 20,953 | - Transnationalism was associated with higher fertility ideals but less so with actual fertility |  |
| Afulani, 2016 (2) | To examine the associations between cross-border ties and separation and the health of sub-Saharan African migrants in France | Survey, cross-sectional | France  Trajectoires et Origines survey (2010)^1^ | In-person interview | Sub-Saharan Africans ages 18-50 (IM,IM2)  L: <5 to 25 years | French,  Unspecified mother tongue | 1980 | - Having children and/or siblings abroad was associated with having a chronic disease among women; having parents abroad was protective - Sending remittances was associated with having a chronic disease among women when she had children abroad - A desire to resettle abroad was associated with having a chronic disease among men |  |
| Alcantara, 2015 (3) | To examine the associations of transnational economic and social ties with past-year major depressive episodes (MDE) | Survey, cross-sectional | United States  National Latino and Asian American Study (2002-2003)^2,3^ | In-person interview | Cuban, Mexican, Puerto Rican, other Latino adults (IM)  L: >5 years for majority | English,  Spanish | 1614 | - Sending remittances was associated with reduced odds of past-year MDE - Return visits were associated with increased odds of past-year MDE |  |
| Alcantara, 2015 (4) | To examine whether transnational ties, social ties, and neighborhood ties are independently associated with the odds of smoking | Survey, cross-sectional | United States  National Latino and Asian American Study (2002-2003)^2,3^ | In-person interview | Cuban, Mexican, Puerto Rican, other Latino adults (IM)  L: ≥ 5 years for majority | English,  Spanish | 1629 | - Return visits were associated with increased odds of being a current smoker - Sending remittances were associated with lower odds of being a current smoker for women |  |
| Ambugo, 2016 (5) | To examine the association between sending remittances and major depressive episodes (MDE) and sadness among legal immigrants in the US | Survey, cross-sectional | United States  Wave 1 of New Immigrant Survey (2006)^4^ | Interview | Latin American, Asian, African, Eastern European/USSR (IM,R, A)  L: <2 years to >11 years | English,  Unspecified | 8236 | - Immigrants who remitted had higher odds of MDE and sadness compared to those who did not remit - The amount remitted was associated with a higher risk of sadness for refugees/asylum seekers compared to economic immigrants |  |
| Amoyaw, 2016 (6) | To examine whether remittance sending has an effect on immigrants’ emotional health over time | Longitudinal Survey | Canada  Longitudinal Survey of Immigrants to Canada (2000-2005)^5^ | Interview | Asian, Middle Eastern, African, South American, Central American, European, North American (IM, R)  L: <5 years | English,  Unspecified | 7269 | - Those remitting shortly after arrival in Canada were more likely to experience emotional health problems compared to non-remitters - Over time sending remittances was associated with improvements in emotional health among females |  |
| Araujo Dawson, 2010 (7) | To examine the associations between racial and ethnic discrimination, and acculturative stress among Dominican immigrants | Survey, cross-sectional | United States | Interview | Dominicans who migrated after the age of 18 (IM)  L: <20 or >=20 years | English,  Spanish | 283 | - Transnationalism was positively correlated with experiences of daily racial discrimination - Experiences of daily racial discrimination and major racist events were significant predictors of acculturative stress |  |
| Araujo Dawson, 2018 (8) | To explore the relationship between transnationalism and perceptions of discrimination among Latino/as | Survey, cross-sectional | United States  Latino National Survey (2006)^6^ | Telephone survey | Dominicans, Cubans, Puerto Ricans (IM,R,IM2)  L: mean of 19.34-34.82 years | English,  Spanish | 1577 | - Transnationalism was a significant predictor of higher perceived discrimination |  |
| Burholt, 2016 (9) | To examine how cultural heritage, social class, social-support networks, and transnational family relationships are associated with cultural identity | Survey, cross-sectional | England and Wales  Inter, Intra-generational and Transnational Caring in Minority communities in England and Wales | In-person interview | Black Caribbean, Black African, Indian, Pakistani, Bangladeshi, Chinese ages 55+ (IM)  L: 4-72 years | English,  Migrant’s mother tongue | 815 | - Transnational relationships were associated with different forms of cultural identity depending on the type of relationship and who the relationship was with (children, relatives, siblings) |  |
| Calvasina, 2015 (10) | To examine the predictors of transnational dental care utilization over a four year period among immigrants to Canada | Longitudinal survey (but analysis was cross-sectional) | Canada  Longitudinal Survey of Immigrants to Canada (2005)^5^ | Interview | South Asian, East Asian, Middle Eastern, African, Latin American, Caribbean, European, ages 20-60 (IM)  L: <5 years | English, Unspecified | 3976 | - Immigrants lacking dental insurance and reporting dental problems were more likely to seek transnational dental care - Being on social assistance was negatively associated with seeking transnational dental care |  |
| Chang, 2018 (11) | To examine factors that influence kirogi mothers’ psychological well-being | Survey, cross-sectional | United States, New Zealand | Online or mailed survey | Korean mothers of a child between the ages of 13 & 18, who are part of a “goose” family (IM)  L: 5-20 years | English, Korean | 153 | - Transnational marital problems were associated with depressive symptoms among Kirogi mothers - Transnational marital satisfaction was associated with life satisfaction |  |
| De Jesus, 2013 (12) | To examine predictors of health care service utilization in Mexico or any other country in Latin America among the US Hispanic population |  | United States  Pew Hispanic Healthcare Survey (2007)^7^ | Telephone survey | Hispanic adults (IM, C, UD)  L: not specified | English  Spanish | 4013 | - Receiving poor or fair quality health care in the US, and having US citizenship/residency increased the likelihood of seeking transnational healthcare - Having health insurance in the US was negatively associated with seeking transnational healthcare |  |
| Dito, 2017 (13) | To examine the consequences of family separations due to international migration on the well-being of Ghanaian migrant parents in the Netherlands | Survey, cross-sectional | Netherlands: Amsterdam, Almere, The Hague  Transnational Child Raising Arrangements between Ghana and the Netherlands (2011)^8^ | In-person interview | Ghanaian parents living transnationally or with their children (IM,UD)  L: mean 10.55 years | English,  Migrants’ mother tongue / dialect | 303 | - Transnational parents had worse health and well-being compared to non-transnational parents, however the effects of transnational parenting on health disappeared when socioeconomic status was considered |  |
| Djundeva, 2020 (14) | To examine how transnational activities shape social support networks of Polish migrants in the Netherlands, and how social support networks are associated with loneliness | Survey, cross-sectional | Netherlands  Families of Poles in the Netherlands Survey (2016)^9^ | Online survey & computer-assisted personal interview | Polish (IM)  L: 1-23 years | Dutch,  Polish | 1131 | - Migrants who sent remittances were more likely to have a kin-focused social support network with low contact frequency |  |
| Flippen, 2015 (15) | To assess the extent and prevalence of reported discrimination among Latin American immigrants across different settings | Survey, cross-sectional | United States: North Carolina  Gender, Migration, and Health among Hispanics study (2012)^10,11^ | In-person interview | Honduran, El Salvadorian, Guatemalan, other Latin American (IM,UD)  L: mean of 4.4 years | English,  Spanish | 1637 | - Among men, intended settlement was positively associated with perceived discrimination - Making visits to the country of origin was associated with discrimination encounters in settings related to housing (e.g. renting) - Those without return intentions were more likely to experience discrimination in public places |  |
| Gelatt, 2013 (16) | To examine whether migrants draw more strongly on comparisons to those in the home country, destination country, or both in evaluating their relative economic and social standing | Survey, cross-sectional | United States  National Latino and Asian American Study (2002-2003)^2,3^ | In-person interview | Vietnamese, Filipino, Chinese, other Asian, Cuban, Mexican, Puerto Rican, other Latin American adults (IM,C,R)  L: <5 to >20 years | English,  Spanish,  Asian languages | 2976 | - There was no evidence that transnational engagement bolsters reliance on referring to the home country as a reference group - There was limited evidence that those with higher transnational involvement rely less on US-based comparisons than those who were not transnationally engaged - Transnationalism was negatively associated with depression in the last month - Transnationalism was positively associated with one saying that they would still migrate if deciding today |  |
| Gherghina, 2020 (17) | To analyze return migration intentions among Romanian migrants | Survey, cross-sectional | Italy, Spain, United Kingdom, Germany, France, United States, Australia, Japan, New Zealand, Singapore, Tonga and others | Online survey | Romanian adult immigrants (IM, UD)  L: not specified | Not specified | 1839 | - Perceived discrimination was associated with having a higher likelihood of return intentions - Having a sense of belonging in the host country was negatively associated with return intentions - Positive assessment of the performance of public institutions in the host country was negatively associated with return intentions |  |
| Greder, 2009 (18) | To detail associations between transnationalism and housing and health risks experienced by rural Latino immigrants in the US | Mixed Methods | United States: California, Iowa, Oregon  Rural Families Speak research project (2004)^12^ | In-person interview | Latin American mothers, predominantly Mexican, El Salvadorian (IM,IM2, UD)  L: <1 to 42 years | English,  Spanish | 78 | - High transnationalism was correlated with housing quality issues and difficulty finding larger housing units - High transnational families were less likely than low transnational families to be knowledgeable about community resources |  |
| Haagsman, 2014 (19) | To investigate how different factors are associated with the quality of parent-child relationships in transnational families | Survey, cross-sectional | Netherlands: Amsterdam, The Hague, Rotterdam, & other areas  Transnational Child-Raising Arrangements between Africa and Europe (2009)^13^ | Interview | Transnational Angolan and Nigerian parents (IM,UD, A)  L of separation from child(ren): 1-20 years | English,  Dutch,  Portuguese | 265 | - The amount of contact with the child abroad was significantly related to the quality of the parent-child relationship - Remittance sending was not significantly related to the quality of the parent-child relationship |  |
| Haagsman, 2015 (20) | To investigate the association between parental subjective well-being and parent-child separation in Angolan and Nigerian migrant parents. | Survey, cross-sectional | Netherlands: Amsterdam, The Hague, Rotterdam, & other areas  Transnational Child-Raising Arrangements between Africa and Europe (2009)^13^ | Interview | Transnational and non-transnational Angolan and Nigerian parents (IM,UD, A)  L: 1-32 years | English,  Dutch,  Portuguese | 607 | - Transnational parenting was negatively associated with happiness - Transnational parenting was negatively associated with self-assessed health for Angolan parents, and also for Nigerian parents if the relationship with children was poor - Transnational parenting was associated with poor mental health among Angolan parents - Transnational parenting was negatively associated with life satisfaction for Nigerian parents, and also for Angolan mothers |  |
| Haagsman, 2018 (21) | To explore the link between transnational family life and job outcomes in Angolan parents | Survey, cross-sectional | Netherlands: Amsterdam, The Hague, Rotterdam, & other areas  Transnational Child-Raising Arrangements between Africa and Europe (2009)^13^ | Interview | Transnational and non-transnational Angolan parents (IM,UD, A)  L: 0-25 years | Dutch,  English,  Portuguese | 255 | - Transnational parents reported changing jobs more often, being less happy, and having more family-to-work conflict than non-transnational parents - Transnational parents who had limited contact with their children had even more job instability |  |
| Horn, 2020 (22) | To investigate the role of transnational ties on Peruvian migrants’ well-being, in particular depression and loneliness | Survey, cross-sectional | Latin America, North America, Europe and others  Worldwide survey on the Peruvian community abroad (WSPCA)^14^ | In-person interview or self-completed survey | Peruvian adults (IM, UD)  L: 1-60 years, mean of 10 years | Not specified | 11,305 | - Those who remit regularly, have return intentions, visit Peru regularly, and purchase more Peruvian articles were more likely to have poor well-being - The odds of having poor well-being were also higher when there were no relatives living in the host country |  |
| Humphries, 2009 (23) | To illustrate the remittance connections maintained by migrant nurses in Ireland | Mixed- methods | Ireland: including 3 large hospitals in the Dublin area | Mail-in survey (Quantitative), Interview (Qualitative) | Nurses from the Philippines, India, Nigeria, South Africa, United States, Australia, Zimbabwe, other countries (IM)  L: 3-9 years for majority | English | 336 (Survey)  21 (Interview) | - More than half of remittance senders reported struggling financially at least occasionally - Those that owned accommodations and intended to remain in Ireland were less likely to send remittances |  |
| Jang, 2017 (24) | To examine factors associated with Korean immigrants’ medical tours to the homeland | Mixed-methods | United States: New York – New Jersey area | Self-completed survey (Quantitative), Interview (Qualitative) | Korean (IM,C, UD)  L: 1-20+ years | English,  Korean (Interview) | 507 (Survey)  120 (Interview) | - Having relatives in Korea, and having frequent contact with relatives were associated with transnational health care seeking |  |
| Johnson, 2008 (25) | To document Sudanese men’s remittance behaviors and attitudes, and to identify the levels of financial and emotional strain experienced | Survey, cross-sectional | Canada: British Columbia | Self-completed questionnaire | Sudanese men (R,C)  L: mean of 3.6 years | English,  Arabic | 172 | - Respondents found it difficult to remit while getting established in Canada, as exhibited by difficulty paying for essentials (ex. rent, monthly bills, food) - Greater emotional financial strain was associated with sending less remittances |  |
| Kemppainien, 2018 (26) | To examine cross-border health care use by Russian immigrants and its associations with social integration and discrimination | Survey, cross-sectional | Finland: 6 urban municipalities  Finnish Migrant Health and Wellbeing Survey (2012)^15^ | In-person interview | Russian (IM, C)  L: <5 to >15 years | Russian,  Finnish | 545 | - Those who were more integrated into Finnish society were less likely to seek cross-border health care - Those that experienced discrimination in Finnish health services, had poor self-rated health or a chronic condition were more likely to seek cross-border health services - Those who experienced economic hardship were less likely to seek cross-border healthcare |  |
| Klok, 2017 (27) | To explore profiles of belonging among older migrants and how these profiles are associated with loneliness | Survey, cross-sectional data used | Netherlands  Longitudinal Aging Study Amsterdam (2011)^16^ | In-person interview | Turkish and Moroccan migrants born between 1948 and 1957 (IM)  L: mean of 36.8 years | Dutch,  Turkish/ Moroccan,  Arabic / Darija,  Tarafit | 461 | - Having feelings of loss with regards to the country of origin was associated with more loneliness |  |
| Mazzucato, 2017 (28) | To investigate the extent to which being in a transnational family affects parents’ well-being in destination countries | Survey, cross-sectional | Netherlands: Amsterdam, the Hague, Rotterdam, Utrecht,  Portugal: Lisbon area  Transnational Child-Raising Arrangements between Africa and Europe (2009)^13^ | Interview | Angolan transnational parents (IM,UD, R, A)  L: mean of 10.62 years (Portugal); mean of 13.77 years (Netherlands) | English,  Dutch,  Portuguese | 605 | - Among Angolan parents in Portugal and the Netherlands, being a transnational parent was associated with worse emotional well-being and lower levels of happiness - Angolan transnational parents in Portugal were also more likely than their non-transnational parent counterparts to report lower levels of life satisfaction and worse self-rated health |  |
| McCabe, 2017 (29) | To examine the health of transnational mothers and whether they differ in demographics, acculturation, acculturation stress, or syndemic factors | Survey, cross-sectional (original study was a randomized trial) | United States: South Florida  Health, Education, Prevention, and Self-care (2012)^17^ | In-person interview | Hispanic mothers, 18-50 years old (IM, IM2)  L: mean of 6.41-12.78 years | English,  Spanish | 425 | - Mothers separated from minor children experienced greater occupational, economic, and immigration stress than mothers who were not separated from their children |  |
| Miranda, 2005 (30) | To examine whether immigrant Latinas separated from their children differ in rates of probable major depression from those who live with their children and from those who do not have children | Survey, cross-sectional | United States: Maryland, Virginia  Women Entering Care study (2003)^18^ | In-person interview | El Salvadorian, Nicaraguan, Guatemalan, Mexican American, Belizean, Costa Rican, and Honduran, South American, Caribbean women with or without children (IM, UD)  L: mean of 6.9 years | English,  Spanish | 5122 | - Rates of depression were highest among those separated from their children, and the odds of having depression were 1.52 times greater for women separated from their children compared to women who lived with their children |  |
| Murphy, 2004 (31) | To develop a quantifiable measure of transnationalism, and to examine transnationalism’s role in mental health outcomes | Survey, cross-sectional | United States: urban New York | In-person or online self-completed survey | West Indians from 12 Caribbean countries (IM)  L: mean of 7.7 years | English | 137 | - Transnationalism was positively correlated with perceived social support, life satisfaction, and depression - Cultural/social and financial/commercial ties and communication with family were positively correlated to perceived social support - Social/family communication was positively correlated with stronger ethnic identity, lower levels of stress and less anxiety - Political/economic activity was positively correlated with more anxiety and depression - Travelling to visit family and friends was positively correlated with more life satisfaction |  |
| Nielsen, 2012 (32) | To investigate the use of transnational healthcare by Turkish immigrants, their descendants, and ethnic Danes | Survey, cross-sectional | Denmark | Computer-assisted telephone interview or online survey | Turkish immigrants (IM, IM2)  Ethnic Danes  (has at least one parent born in Denmark with Danish citizenship)(NM)  L: not specified | Danish,  Turkish | 1999 | - Turkish immigrants made the highest use of transnational healthcare, followed by Turkish descendants, then ethnic Danes - Socio-economic position and having health symptoms had a fairly explanatory effect on transnational healthcare use |  |
| Pannetier, 2017 (33) | To understand the role of transnational ties in mental health among sub-Saharan African migrants in France | Survey, cross-sectional | France: Paris  Parcours Study (2016)^19^ | In-person interview | Sub-Saharan African (mostly Western and Central African, including Côte-d’Ivoire, Cameroon, Mali, Democratic Republic of the Congo, Senegal) Immigrants ages 18-59, living with HIV or Chronic Hepatitis B, or free from these infections (IM, UD)  L: median of 13 years (women), 12 years (men) | French,  Migrants’ mother tongue | 2468 | - Cross-border separation from a child, and having transnational social support were not significantly associated with anxiety and depression however, having social connections both in the home country and the host country, was associated with reduced levels of depression and anxiety |  |
| Razum, 2019 (34) | To examine whether transnational ties contribute to health satisfaction in immigrants in Germany | Survey, cross-sectional | Germany  German Socio-Economic Panel (2014)^20,21^ | Interview | Turkish, Former Yugoslavia, Italian, other regions (IM)  L: 5-40+ years | German ?,  Migrants’ mother tongue (?) | 1589 | - Transnational ties had no significant associations with health satisfaction after adjusting for control variables |  |
| Samari, 2016 (35) | To examine whether cross-border ties are protective for mental health and whether they influence psychological distress and happiness in several immigrant generations | Survey, cross-sectional | United States: Detroit  Detroit Arab American Study (2006)^22^ | In-person interview | Self-identifying Arab or Chaldean migrants and descendants (IM,C,IM2, IM3+)  L: not specified | Arabic,  English | 896 | - The types of transnational ties maintained by participants differed by nativity - Migrants and descendants with more positive cross-border attitudes and social ties had higher odds of psychological distress, whereas immigrants that participated in local organizations with ties to the Arab world had lower psychological distress - First generation migrants who consumed media and had positive cross-border attitudes had lower levels of happiness |  |
| Shooshtari, 2014 (36) | To examine the impact of sending remittances on housing and living conditions and overall health status | Longitudinal survey | Canada  Longitudinal Survey of Immigrants to Canada (2005)^5^ | Interview | Filipino/as who arrived in Canada between 2000-2001, aged 15 or older at arrival (IM, R?)  L: <5 years | English,  Unspecified | 529 | - Remittances had no independent effect on the overall health status of recent immigrants who remitted at 6 months or 2 years after arrival - Those remitting at 6 months had significantly increased odds of renting than owning housing |  |
| Snel, 2006 (37) | To examine how various migrant groups’ patterns of transnationalism relate to their integration into Dutch society | Survey, cross-sectional | Netherlands | In-person interview | Moroccan, Dutch Antillean, Iraqi, Yugoslavian, Japanese, American (IM,IM2,R)  L: measured but not specified | Dutch,  Migrant’s mother tongue | 300 | - Participation in socio-cultural activities in the home country was negatively associated with identifying with the host country - Having transnational economic/professional ties and identifying with a transnational diaspora were positively associated with identifying with the host country |  |
| Su, 2012 (38) | To examine whether acculturation affects the use of cross-border health services for Mexican American | Survey, cross-sectional | United States: Texas  Cross-border Utilization of Health Care Survey (2008) | Telephone interview | Self-identifying Hispanics (Mexican) ages 18 or older (IM,C,UD,IM2,IM3+)  L: not specified | English,  Spanish | 966 | - The most commonly utilized transnational health service was dental care, then medication purchase, then doctor visits - As generation in the US progressed, the probability of seeking transnational health services was steadily smaller - Lack of insurance and poor quality care may explain transnational health service use |  |
| Torres, 2013 (39) | To examine the extent to which continued cross-border ties contribute to overall health status for young 1.5 or 2^nd^ generation Latino immigrants | Survey, cross-sectional | United States: 5 counties in Los Angeles  Study of Immigration and Intergenerational Mobility in Metropolitan Los Angeles (2004)^23^ | Telephone survey | Mexican, Mexican-American, Central American young adults who immigrated before age 15 or have at least one parent born abroad (IM,IM2,C,UD)  L: not specified | English,  Spanish | 1268 | - Among 1.5 generation immigrant young adults having a close relative abroad was positively associated with self-rated health - Having a parent who did extended visits abroad was negatively associated with self-rated health for 1.5 and 2^nd^ generation young adults - Having parents who remitted was positively associated with self-rated health for 1.5 and 2^nd^ generation young adults |  |
| Torres, 2016 (40) | To examine the potential associations between cross-border ties and psychological distress in Latino migrants living in the US | Survey, cross-sectional | United States  National Latino and Asian American survey (2002-2003)^2,3^ | In-person interview | Cuban, Mexican, Puerto Rican, other Latino/as (IM,UD,C)  L: >20 years or <20 years | English,  Spanish | 1573 | - Sending remittances was shown to be protective for psychological distress among Cuban migrants - Difficulty visiting home was associated with more psychological distress among Puerto Rican migrants |  |
| Torres, 2016 (41) | To examine the effects of cross-border ties on trajectories of depression in immigrant and US-born Latino adults | Longitudinal survey | United States: Northern California  Sacramento Area Latino Study on Aging (2003)^24^ | Mail, phone or in person survey (self-completed?) | Foreign and US-born Latino/as (primarily Mexican) ages 60 and older (IM,IM2)  L: mean > 30 years | English,  Spanish | 1270 | - The odds of depression for Latina immigrants were significantly greater if they reported social ties or travelling to Latin America - The odds of depression for Latino men (immigrant and US born) were significantly lower if they reported travelling to Latin America |  |
| Torres, 2018 (42) | To estimate the associations between an indicator of cross-border social ties and 3 inflammatory markers in older Latino adults | Longitudinal survey | United States: Northern California  Sacramento Area Latino Study on Aging (2003)^24^ | In-person interview | Foreign and US-born Mexican & Central Americans aged 60-101 (NM,IM, IM2+, C)  L: Not specified | English,  Spanish | 1786 | - Immigrants with any cross-border ties had lower levels of inflammatory markers at baseline and at the 5-year follow-up compared to US-born respondents with no cross-border ties |  |
| Torres, 2019 (43) | To examine the independent associations between cross-border ties and alcohol use among US migrants | Survey, cross-sectional | United States  National Latino and Asian American Survey (2002-2003)^2,3^ | In-person interview | Chinese, Filipino, Vietnamese, other Asian, Mexican, Cuban, Puerto Rican, and other Latino (IM)  L: age at migration ranged from < 12 to > 35 years old | English,  Spanish | 3271 | - For Latin American-origin women, cross-border visits were associated with increased risk for alcohol use among those who migrated early in life - Asian-origin men and women who migrated as adults and who had contacts with family and friends had the lowest probabilities of alcohol use within the last year |  |
| Van den Broek, 2017 (44) | To assess feelings of loneliness among Polish migrants in the Netherlands and whether they vary by presence and location of partners and offspring. | Survey, cross-sectional | Netherlands  Families of Poles in the Netherlands (2016)^9^ | Online survey & computer-assisted personal interview | Polish-born adults who have at least one Polish parent, ages 18-49 (IM)  L: < 3 years to >7 years | Dutch,  Polish | 1129 | - For men, having a partner living abroad was associated with higher levels of loneliness |  |
| Vaquera, 2011 (45) | To examine the relationship between transnational activities and subjective well-being in immigrants | Survey, cross-sectional | United States: South Florida | Telephone survey | Predominantly Cuban, Colombian, Haitian, Other Hispanic, Other non-Hispanic (IM,C)  L: <1 year to >80 years, mean of 21.94 years | English,  Spanish, Haitian,  Migrants’ mother tongue? | 1268 | - Sending remittances, frequent visits back home and thinking about moving back were associated with lower levels of emotional well-being - Having confidantes and making visits back home non-frequently were positively associated with emotional well-being - Making visits non-frequently was positively associated with life satisfaction, however thinking about moving back and considering the home country as “home” were negatively associated with life satisfaction |  |
| Wang, 2015 (46) | To capture the experience of Korean immigrants in seeking and receiving healthcare locally and transnationally | Mixed methods | Canada: (Toronto Qualitative)  Canadian Community Health Survey by Statistics Canada (2005-2010)^25^ | Telephone or computer assisted interview (Quantitative),  Focus group + questionnaire (Qualitative) | Korean (IM)  L: <5 years to >20 years | Korean (Focus group, questionnaire)  English  (Survey) | 351 (Survey)  &  54  (Focus group) | - Approximately one-third of focus group participants or their family members used transnational healthcare services - Long wait times, care not meeting expectations, and not having dental insurance were some of the reasons reported for using transnational healthcare |  |
| White, 2019 (47) | To examine the health and emotional well-being of Nigerian migrant parents living in Ireland and the Netherlands | Survey, cross-sectional | Ireland and the Netherlands  Transnational Child-Raising Arrangements between Africa and Europe (2009)^13^ | In-person interview | Nigerian adults with children transnationally or non-transnationally (IM,UD, A)  L: mean of 6.93-8.53 | Dutch  English | 609 | - Transnational parents in both locations reported lower levels of life satisfaction, and lower emotional well-being, compared to non-transnational parents, however these outcomes were largely explained by social, migration and economic factors |  |

^++^ NM = non-migrant, IM = immigrant, R = refugee, UD = undocumented/irregular migrant, C = citizen of destination country, A = asylum-seeker, ? = not specified/unclear, IM2 = 2^nd^ generation immigrant, IM2+= 2^nd^ generation immigrant and more, IM3+ = 3^rd^ generation immigrant or more

^+^ ***References for the data/sample source or parent project***:

^1^ Beauchemin, C., Hamel, C., & Simon, P. (2010). *Trajectories and origins: Survey on population diversity in France. Working Paper No. 168.1*. Institut National d’Etudes Demographiques (INED).

^2^ Alegria, M., Takeuchi, D., Canino, G., Duan, N., Shrout, P., Meng, X.-L.,…Gong, F. (2004). Considering context, place and culture: The National Latino and Asian American Study. *International Journal of Methods in Psychiatric Research, 13,* 208-220.

^3^ Heeringa, S. G., Wagner, J., Torres, M., Duan, N., Adams, T., & Berglund, P. (2004). Sample designs and sampling methods for the Collaborative Psychiatric Epidemiology Studies (CPES). *International Journal of Methods in Psychiatric Research, 13,* 221–240.

^4^ Jasso, G., Massey, D. S., Rosenzweig, M. R., & Smith, J. P. (2006). *The New Immigrant Survey 2003 round 1 (NIS-2003-1)*. Public release data 2006.

^5^ Statistics Canada. *Longitudinal Survey of Immigrants to Canada*. Available at: <https://crdcn.org/datasets/lsic-longitudinal-survey-immigrants-canada>

^6^ Fraga, L.R., Garcia, J.A., Hero, R., Jones-Correa, M., Martinez-Ebers, V., & Segura, G.M. (2013). *Latino National Survey (LNS), 2006*. Inter-university Consortium for Political and Social Research [distributor]. Available at: <https://doi.org/10.3886/ICPSR20862.v6>

^7^ Pew Hispanic Center. (2007). *Pew Hispanic Center: 2007 Hispanic Healthcare Survey* *(Version 2)* [Dataset]. Cornell University, Ithaca, NY: Roper Center for Public Opinion Research. doi:10.25940/ROPER-31095908

^8^ Effects of Transnational Child Raising Arrangements on Life-Chances of Children, Migrant Parents and Caregivers between Africa and The Netherlands. TCRA Ghana. Available at: <https://fasos-research.nl/tcra/tcra-ghana/>

^9^ Karpinska, K., Dykstra, P. A., & Fokkema, T. (2016). Families of Poles in the Netherlands (FPN) Survey. Wave 1. *DANS.* doi:10.17026/danszep-et7y

^10^ Flippen, C., & Parrado, E. (2012). Forging Hispannic Communities in New Destinations: A Case Study of Durham, North Carolina. *City & Community, 11*, 1-30.

^11^ Parrado, E.A, McQuiston, C. & Flippen, C. (2005). Participatory Survey Research: Integrating Community Collaboration and Quantitative Methods for the Study of Gender and HIV Risks among Hispanic Migrants. *Sociological Methods & Research*, *34*, 204-39.

^12^ Bauer, J. W. (2004). *Basebook Report: Low income rural families: Tracking their well-being and functioning in the context of welfare reform. North Central Region, Multi State Project NC223*. Unpublished report.

^13^ Effects of Transnational child-raising arrangements on life-chances of children, migrant parents and caregivers between Africa and Europe TCRAf-Eu. Available at: <https://fasos-research.nl/tcra/tcraf-eu/>

^14^ Ministerio de Relaciones Exteriores, Instituto Nacional de Estadística e Informática- INEI, Organización Internacional para las Migraciones- OIM. (2013). *Resultado de la Primera Encuesta Mundial a la Comunidad peruana en el exterior 2012*. Perú.

^15^ Castaneda, A., Rask, S., Koponen, P., Molsa, M., Koskinen, S., editors. (2012). Migrant health and wellbeing. In *A Study of Persons of Russian, Somali and Kurdish Origin in Finland.* THL, Helsinki. Available at: http://urn.fi/URN:ISBN: 978-952-245-739-4

^16^ Huisman, M., Poppelaars, J., Van der Horst, M., Beekman, A. T., Brug, J., Van Tilburg, T. G., & Deeg, D. J. (2011). Cohort profile: the longitudinal aging study Amsterdam. *Int J Epidemiol 40*, 868-876.

^17^ Peragallo, N., Deforge, B., O’Campo, P. Lee, S.M., Kim, Y.J., Cianlelli, R., & Ferrer, L. (2005). A randomized clinical trial of an HIV risk reduction intervention among low-income Latina women. *Nursing Research*, *54,* 108-118.

^18^ Miranda, J., Chun, J. Y., Green B. L….et al. (2003). Treating depression in predominantly low-income young minority women: a randomized controlled trial. *JAMA 290:*57-65.

^19^ Desgrées-du-Lou, A., Pannetier, J., Ravalihasy, A., Le Guen, M., Gosselin, A., Panjo, H., & PARCOURS Study Group (2016). Is hardship during migration a determinant of HIV infection? Results from the ANRS PARCOURS study of sub-Saharan African migrants in France. *AIDS* *(London, England), 30*, 645-656.

^20^ Gerstorf, S., & Schupp, J. (2014). *SOEP wave report 2013*. DIW, Berlin.

^21^ Wagner, G.G., Frick, J.R., & Schupp, J. (2007). The German socio-economic panel study (SOEP)- scope evolution and enhancements. *Schmollers Jahr, 127*, 139-170.

^22^ Baker, W., Stockton, R., Howell, S., Jamal, A., Lin, A. C., Shryock, A., et al. (2006). *Detroit Arab American Study (DAAS), 2003*. Inter-University Consortium for Political and Social Research (ICPSR) [distributor].

^23^ Rumbaut, R.G., Bean, F. D., Chavez, L. R., Lee, J., Brown, S. K., DeSipio, L., et al. (2004). *Immigration and intergenerational mobility in metropolitan Los Angeles.* Ann Arbor, MI: Inter-University Consortium for Political and Social Research.

^24^ Haan, M., Aiello, A., Gonzalez, H., Hinton, L., Jagust, B., Miller, J., … Seavey, W. (2018). *Sacramento Area Latino Study on Aging (SALSA Study), 1996-2008: Semi-Annual Phone Call Data*. Ann Arbor, MI: Inter-university Consortium for Political and Social Research [distributor]. Available at: <https://doi.org/10.3886/ICPSR29321.v2>

^25^ Statistics Canada. *Canadian Community Health Survey*. Available at: https://www23.statcan.gc.ca/imdb/p2SV.pl?Function=getSurvey&SDDS=3226

**Appendix II: Summary of the operationalization of the transnationalism measures**

**Table A.** Social ties

| Reference  # | Contact with family / friends in home country | | | | Return visits | | | | | | Presence of confidants / partner | | |
| --- | --- | --- | --- | --- | --- | --- | --- | --- | --- | --- | --- | --- | --- |
|  | Since arrival / in general | Freq | Method of communication | At least once | | | In the last year / 2 years | Freq / # of visits per year | Difficulty making return visits | In the home country | | Ratio of confidants in destination to home country | Partner from home country |
| (1) | X |  |  | X | |  | |  |  |  | |  |  |
| (2) | X |  |  | X | |  | |  |  |  | |  |  |
| (3) |  |  |  |  | | X | |  |  |  | |  |  |
| (4) |  |  |  |  | | X | |  |  |  | |  |  |
| (7) | X |  |  | X | |  | |  |  |  | |  |  |
| (8) |  |  |  |  | |  | | X |  |  | |  |  |
| (9) |  | X | X | X | |  | |  |  |  | |  |  |
| (14) |  |  |  |  | |  | | X |  |  | | X |  |
| (15) |  |  |  | X | |  | |  |  | X | |  | X |
| (16) |  |  |  |  | | X | |  |  |  | |  |  |
| (18) |  | X |  |  | |  | | X |  |  | |  |  |
| (22) |  |  |  |  | |  | | X |  | X | |  |  |
| (24) |  | X |  |  | |  | |  |  | X | |  |  |
| (27) |  | X |  | X | |  | |  |  |  | |  |  |
| (31) | X |  |  | X | |  | |  |  |  | |  |  |
| (33) |  |  |  |  | |  | |  |  | X | |  |  |
| (34) | X |  |  |  | | X | |  |  | X | |  |  |
| (35) | X |  |  | X | |  | |  |  | X | |  | X |
| (37) | X |  |  | X | |  | |  |  |  | |  |  |
| (39) |  |  |  | X^a^ | |  | |  |  | X | |  |  |
| (40) |  |  |  |  | |  | |  | X |  | |  |  |
| (41) |  | X |  |  | |  | | X |  |  | |  |  |
| (42) |  | X |  |  | |  | |  |  |  | |  |  |
| (43) | X |  |  |  | |  | | X |  |  | |  |  |
| (45) |  | X |  | X | |  | |  |  |  | | X |  |
| (46) | X |  |  |  | | X | |  |  |  | |  |  |

^a^ Also examined whether parents of second generation migrants visited home when they were children, and if they accompanied them

**Table B.** Measures related to transnational family status

| Reference # | Cross-border separation with partner/spouse | Transnational parenting status | Parent-child relationship (as measured by frequency of contact with child) | Remittances | Location of confidant |
| --- | --- | --- | --- | --- | --- |
| (11) | X |  |  |  |  |
| (13) |  | X |  |  |  |
| (19) |  |  | X | X |  |
| (20) |  | X | X |  |  |
| (21) |  | X | X |  |  |
| (28) |  | X |  |  |  |
| (29) |  | X |  |  |  |
| (30) |  | X |  |  |  |
| (33) |  | X^a^ |  |  | X |
| (44) | X | X |  |  |  |
| (47) |  | X |  |  |  |

^a^Where child is under 18 years-old

**Table C.** Cultural ties

| Reference  # | Religiosity | | | Use of home country language | Participation in cultural activities | | | | | Media consumption | | | |
| --- | --- | --- | --- | --- | --- | --- | --- | --- | --- | --- | --- | --- | --- |
|  | Religious service attendance per year | Involvement in religious organization | Church attended by other compatriots |  | Cultural festivities of home country | Cultural clubs related to home country | Sponsor entertainers from home country to perform in destination country | Attends meetings with primarily compatriots | | Newspaper | Internet | TV | Radio |
| (1) |  |  |  |  |  |  |  | | X | X | X | X | X |
| (2) |  |  |  |  |  |  |  | | X | X | X | X | X |
| (6) |  |  |  |  |  | X |  | |  |  |  |  |  |
| (7) |  |  | X |  | X | X | X | |  |  |  |  |  |
| (14) | X |  |  |  |  |  |  | |  |  |  |  |  |
| (18) |  |  |  | X  (at home) |  |  |  | |  |  |  |  |  |
| (22) |  |  |  |  |  |  |  | |  |  | X |  |  |
| (31) |  |  | X |  | X | X | X | |  |  |  |  |  |
| (34) |  |  |  | X  (in general) |  |  |  | |  | X |  |  |  |
| (35) |  | X |  | X | X | X |  | |  | X | X | X | X |
| (37) |  |  |  |  | X | X |  | | X | X |  |  |  |
| (45) |  |  |  |  | X |  |  | |  |  |  |  |  |
| (46) |  |  |  |  |  |  |  | |  | X | X | X |  |

**Table D.** Economic ties

| Reference # | Remittances | | | | | Business / Financial Assets in home country | | | | Donations / Funding |
| --- | --- | --- | --- | --- | --- | --- | --- | --- | --- | --- |
|  | Time | | In the past year | Recipient | Amount sent / Avg amount sent / Remittance burden (% of income sent) | Owner / investor in a business / conducts trade with home country | Own land / property | Buy / import supplies from home country | Visits home country for business purposes | Project funding |
|  | Since arrival | Frequency |  |  |  |  |  |  |  |  |
| (1) |  |  | X |  |  | X | X |  |  | X |
| (2) |  |  | X |  |  | X | X |  |  | X |
| (3) |  |  |  |  | X |  |  |  |  |  |
| (4) | X |  |  |  |  |  |  |  |  |  |
| (5) | X |  |  |  | X |  |  |  |  |  |
| (6) | X |  |  |  | X |  |  |  |  |  |
| (7) | X |  |  |  |  | X | X | X |  |  |
| (8) |  | X |  |  |  |  | X |  |  |  |
| (9) | X^b^ |  |  |  |  |  |  |  |  |  |
| (14) | X |  |  |  |  |  |  |  |  |  |
| (16) | X |  |  |  |  |  |  |  |  |  |
| (18) | X |  |  |  |  |  |  |  |  |  |
| (19) |  | X  (monthly) |  |  |  |  |  |  |  |  |
| (22) |  | X |  |  |  |  |  | X |  |  |
| (23) | X |  |  | X | X |  |  |  |  |  |
| (25) |  | X |  |  | XX |  |  |  |  |  |
| (28) |  |  |  |  |  |  | X |  |  |  |
| (31) | X |  |  |  |  | X | X | X |  |  |
| (34) | X |  |  |  |  |  |  |  |  |  |
| (36) | X |  |  |  | X |  |  |  |  |  |
| (37) | X |  |  |  |  | XX | X |  | X | X |
| (39) | X^a^ |  |  |  |  |  |  |  |  |  |
| (40) | X |  |  |  |  |  |  |  |  |  |
| (45) |  | X |  |  |  | X |  |  |  |  |
| (46) |  |  | X^b^ |  |  |  | X |  |  |  |
| (47) |  |  |  |  |  | X | X |  |  |  |

^a^ Examined whether parents sent remittances in addition to respondents themselves

^b^ Also examined whether respondents received remittances from home country

**Table E.** Political ties

| Reference # | Interest in national politics | Participation in associations / politics in home country | Voting pre- or post-migration | Monetary donation to a candidate / party from home country | Participation in demonstrations related to home country |
| --- | --- | --- | --- | --- | --- |
| (1) | X |  |  |  |  |
| (2) | X |  |  |  |  |
| (7) | X | X |  |  |  |
| (8) |  | X | X | X |  |
| (31) | X | X |  | X |  |
| (35) | X^a^ |  |  |  |  |
| (37) | X | X |  |  | X |

^a^ Item: “Supports Palestine”

**Table F.** Transnational attitude & identity

| Reference # | Primary Residence | Citizenship in home country | Return intentions | Identification with home country heritage | Feelings of loss / attachment regarding home country |
| --- | --- | --- | --- | --- | --- |
| (8) |  |  | X |  |  |
| (12) |  |  |  | X |  |
| (14) |  |  | X |  |  |
| (15) |  |  | X |  |  |
| (16) | X | X |  |  |  |
| (17) |  |  | X |  |  |
| (22) |  |  | X |  |  |
| (27) |  |  | X |  | X |
| (34) |  |  |  |  | X |
| (35) |  |  |  |  | X |
| (37) |  |  |  | X |  |
| (45) |  |  | X |  | X |

**Table G.** Transnational healthcare use

| Reference # | Time | | Type of medical care | | | | | | |
| --- | --- | --- | --- | --- | --- | --- | --- | --- | --- |
|  | Since arrival | Frequency | Physician (e.g. GP) | | Specialist | | In-patient care | Medication | Dental care |
| (10) |  |  |  |  | |  | |  | X |
| (12) |  | X |  |  | |  | |  |  |
| (24) |  | X |  |  | |  | |  |  |
| (26) |  |  | X^b^ |  | |  | |  |  |
| (27) | X |  |  |  | |  | |  |  |
| (32) ^c^ |  |  | X | X | | X | | X | X |
| (38) |  |  | X |  | | X | | X | X |

^a^ Ever within the last 12 months

^b^ Within the last year

^c^ Examined use of multiple health services within the last year

**References for Appendices I and II**

1. Afulani PA, Asunka J. Socialization, adaptation, transnationalism, and the reproductive behavior of sub-Saharan African migrants in France. Population Research and Policy Review. 2015;34(4):561-92.

2. Afulani PA, Torres JM, Sudhinaraset M, Asunka J. Transnational ties and the health of sub-Saharan African migrants: The moderating role of gender and family separation. Social Science & Medicine. 2016;168:63-71.

3. Alcántara C, Chen C-N, Alegría M. Transnational ties and past-year major depressive episodes among Latino immigrants. Cultural Diversity & Ethnic Minority Psychology. 2015;21(3):486.

4. Alcántara C, Molina KM, Kawachi I. Transnational, social, and neighborhood ties and smoking among Latino immigrants: does gender matter? American Journal of Public Health. 2015;105(4):741-9.

5. Ambugo EA, Yahirun JJ. Remittances and risk of major depressive episode and sadness among new legal immigrants to the United States. Demographic Research. 2016;34:243-58.

6. Amoyaw JA, Abada T. Does helping them benefit me? Examining the emotional cost and benefit of immigrants' pecuniary remittance behaviour in Canada. Social Science & Medicine. 2016;153:182-92.

7. Araújo Dawson B, Panchanadeswaran S. Discrimination and acculturative stress among first-generation Dominicans. Hispanic Journal of Behavioral Sciences. 2010;32(2):216-31.

8. Araujo Dawson B, Suárez ZE. How does transnationalism affect the perceptions of discrimination among Dominicans, Puerto Ricans and Cubans? Journal of Human Behavior in the Social Environment. 2018;28(2):162-76.

9. Burholt V, Dobbs C, Victor C. Transnational relationships and cultural identity of older migrants. GeroPsych. 2016; 29(2):57-69.

10. Calvasina P, Muntaner C, Quiñonez C. Transnational dental care among Canadian immigrants. Community dentistry & oral epidemiology. 2015;43(5):444-51.

11. Chang ES. Kirogi Women’s Psychological Well-Being: The relative contributions of marital quality, mother–child relationship quality, and youth’s educational adjustment. Journal of Family Issues. 2018;39(1):209-29.

12. De Jesus M, Xiao C. Cross-border health care utilization among the Hispanic population in the United States: implications for closing the health care access gap. Ethnicity & health. 2013;18(3):297-314.

13. Dito BB, Mazzucato V, Schans D. The effects of transnational parenting on the subjective health and well‐being of Ghanaian migrants in the Netherlands. Population, Space and Place. 2017;23(3):e2006.

14. Djundeva M, Ellwardt L. Social support networks and loneliness of Polish migrants in the Netherlands. Journal of Ethnic and Migration studies. 2020;46(7):1281-300.

15. Flippen CA, Parrado EA. Perceived discrimination among Latino immigrants in new destinations: The case of Durham, North Carolina. Sociological Perspectives. 2015;58(4):666-85.

16. Gelatt J. Looking down or looking up: Status and subjective well-being among Asian and Latino immigrants in the United States. International Migration Review. 2013;47(1):39-75.

17. Gherghina S, Plopeanu A-P, Necula C-V. The Impact of Socio-Cultural Integration on Return Intentions: Evidence from a Survey on Romanian Migrants. Journal of Immigrant & Refugee Studies. 2020;18(4):515-28.

18. Greder K, Sano Y, Cook CC, Garasky S, Ortiz L, Ontai L. Exploring relationships between transnationalism and housing and health risks of rural Latino immigrant families. Family & Consumer Sciences Research Journal. 2009;38(2):186-207.

19. Haagsman K, Mazzucato V. The quality of parent–child relationships in transnational families: Angolan and Nigerian migrant parents in The Netherlands. Journal of Ethnic and Migration studies. 2014;40(11):1677-96.

20. Haagsman K, Mazzucato V, Dito BB. Transnational families and the subjective well-being of migrant parents: Angolan and Nigerian parents in the Netherlands. Ethnic and Racial Studies. 2015;38(15):2652-71.

21. Haagsman K. Do transnational child-raising arrangements affect job outcomes of migrant parents? Comparing Angolan parents in transnational and nontransnational families in the Netherlands. Journal of family issues. 2018;39(6):1498-522.

22. Horn V, Fokkema T. Transnational ties: Resource or stressor on Peruvian migrants' well‐being? Population, Space and Place. 2020;26(8):e2356.

23. Humphries N, Brugha R, McGee H. Sending money home: a mixed-methods study of remittances by migrant nurses in Ireland. Human Resources for Health. 2009;7(1):1-12.

24. Jang SH. Factors associated with Korean immigrants' medical tourism to the homeland. American journal of health behavior. 2017;41(4):461-70.

25. Johnson PJ, Stoll K. Remittance patterns of southern Sudanese refugee men: Enacting the global breadwinner role. Family Relations. 2008;57(4):431-43.

26. Kemppainen L, Kemppainen T, Skogberg N, Kuusio H, Koponen P. Immigrants ‘use of health care in their country of origin: the role of social integration, discrimination and the parallel use of health care systems. Scandinavian journal of caring sciences. 2018;32(2):698-706.

27. Klok J, van Tilburg TG, Suanet B, Fokkema T, Huisman M. National and transnational belonging among Turkish and Moroccan older migrants in the Netherlands: protective against loneliness? European Journal of Ageing. 2017;14(4):341-51.

28. Mazzucato V, Dito BB, Grassi M, Vivet J. Transnational parenting and the well‐being of Angolan migrant parents in Europe. Global Networks. 2017;17(1):89-110.

29. McCabe BE, Mitchell EM, Gonzalez-Guarda RM, Peragallo N, Mitrani VB. Transnational Motherhood: Health of Hispanic Mothers in the United States Who Are Separated From Children. Journal of Transcultural Nursing. 2017;28(3):243-50.

30. Miranda J, Siddique J, Der-Martirosian C, Belin TR. Depression among Latina immigrant mothers separated from their children. Psychiatric Services. 2005;56(6):717-20.

31. Murphy EJ, Mahalingam R. Transnational ties and mental health of Caribbean immigrants. Journal of Immigrant Health. 2004;6(4):167-78.

32. Nielsen SS, Yazici S, Petersen SG, Blaakilde AL, Krasnik A. Use of cross-border healthcare services among ethnic Danes, Turkish immigrants and Turkish descendants in Denmark: a combined survey and registry study. BMC Health Services Research. 2012;12(390):1-10.

33. Pannetier J, Lert F, Roustide MJ, Du Loû AD. Mental health of sub-Saharan African migrants: the gendered role of migration paths and transnational ties. SSM-population health. 2017;3:549-57.

34. Razum O, Breckenkamp J, Fauser M. Transnational ties, endowment with capital, and health of immigrants in Germany: cross-sectional study. Journal of Public Health. 2019;27(4):507-17.

35. Samari G. Cross-border ties and Arab American mental health. Social Science & Medicine. 2016;155:93-101.

36. Shooshtari S, Harvey CD, Ferguson E, Heinonen T, Khan S. Effects of remittance behavior on the lives of recent immigrants to Canada from the Philippines: A population-based longitudinal study. Journal of Family and Economic Issues. 2014;35(1):95-105.

37. Snel E, Engbersen G, Leerkes A. Transnational involvement and social integration. Global networks. 2006;6(3):285-308.

38. Su D, Wang D. Acculturation and cross-border utilization of health services. Journal of Immigrant and Minority Health. 2012;14(4):563-9.

39. Torres JM. Cross-border ties and self-rated health status for young Latino adults in Southern California. Social Science & Medicine. 2013;81:79-86.

40. Torres JM, Alcántara C, Rudolph KE, Viruell-Fuentes EA. Cross-border ties as sources of risk and resilience: do cross-border ties moderate the relationship between migration-related stress and psychological distress for latino migrants in the United States? Journal of health and social behavior. 2016;57(4):436-52.

41. Torres JM, Lee A, González HM, Garcia L, Haan MN. A longitudinal analysis of cross-border ties and depression for Latino adults. Social Science & Medicine. 2016;160:111-9.

42. Torres JM, Epel ES, To TM, Lee A, Aiello AE, Haan MN. Cross-border ties, nativity, and inflammatory markers in a population-based prospective study of Latino adults. Social Science & Medicine. 2018;211:21-30.

43. Torres JM, Ro A, Sudhinaraset M. Reconsidering the Relationship between Age at Migration and Health Behaviors among US Immigrants: The Modifying Role of Continued Cross-border Ties. Advances in Medical Sociology. 2019;19:17-45.

44. van den Broek T, Grundy E. Loneliness among Polish migrants in the Netherlands: The impact of presence and location of partners and offspring. Demographic Research. 2017;37:727-42.

45. Vaquera E, Aranda E. The multiple dimensions of transnationalism: Examining their relevance to immigrants' subjective well-being. Journal of Social Research & Policy. 2011;2(2):47-72.

46. Wang L, Kwak M-J. Immigration, barriers to healthcare and transnational ties: A case study of South Korean immigrants in Toronto, Canada. Social Science & Medicine. 2015;133:340-8.

47. White A, Dito BB, Veale A, Mazzucato V. Transnational migration, health and well-being: Nigerian parents in Ireland and the Netherlands. Comparative Migration Studies. 2019;7(1):1-26.
